# Design, Synthesis, and Biological Effect Studies of Novel Benzofuran–Thiazolylhydrazone Derivatives as Monoamine Oxidase Inhibitors

**DOI:** 10.1021/acsomega.3c07703

**Published:** 2024-02-29

**Authors:** Derya Osmani̇ye, Begüm Nurpelin Sağlik, Serkan Levent, Ulviye ACAR Çevi̇k, Sinem Ilgin, Leyla Yurttaş, Yusuf Özkay, Ahmet Cagri Karaburun, Zafer Asım Kaplancikli, Nalan Gundogdu-Karaburun

**Affiliations:** † Department of Pharmaceutical Chemistry, Faculty of Pharmacy, Anadolu University, 26470 Eskişehir, Turkey; ‡ Central Research Laboratory (MERLAB), Faculty of Pharmacy, Anadolu University, 26470 Eskişehir, Turkey; § Department of Pharmaceutical Toxicology, Faculty of Pharmacy, Anadolu University, 26470 Eskişehir, Turkey

## Abstract

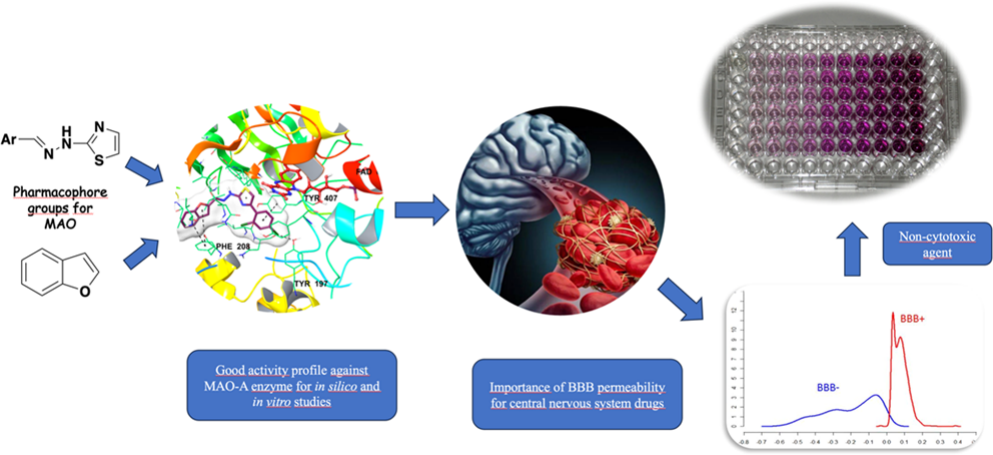

In recent studies, monoamine oxidase (MAO) inhibitory effects of various thiazolylhydrazone derivatives have been demonstrated. Within the scope of this study, 12 new compounds containing thiazolylhydrazone groups were synthesized. The structures of the obtained compounds were elucidated by ^1^H NMR, ^13^C NMR, and high-resolution mass spectrometry (HRMS) methods. The inhibitory effects of the final compounds on MAO enzymes were investigated by means of in vitro methods. In addition to enzyme inhibition studies, enzyme kinetic studies of compounds with high inhibitory activity were examined, and their effects on substrate–enzyme relations were investigated. Additionaly, cytotoxicity tests were carried out to determine the toxicities of the selected compounds, and the compounds were found to be nontoxic. The interactions of the active compound with the active site of the enzyme were characterized by in silico methods.

## 1 Introduction

Monoamine oxidases (MAOs, EC 1.4.3.4) are a class of enzymes including covalently bound redox cofactor flavin adenine dinucleotide (FAD).^1^ Located in the outer mitochondrial membrane of mammalian tissues, the enzyme is distributed to various cells in both the central and peripheral nervous systems. It catalyzes the oxidative deamination of endogenous and exogenous amines to their respective aldehydes enzymatically. It contains two isoforms named MAO-A and MAO-B. MAO-A inhibitors are used to treat depression. The reason for this use is that MAO-A metabolizes serotonin in the central nervous system. MAO-B inhibitors are used to treat Parkinson’s disease (PD). The fact that MAO-B is responsible for dopamine metabolism explains its role in treatment.^2,3^

MAOs have attracted the interest of medicinal chemists since the 1950s. Iproniazid was the first monoamine oxidase inhibitor (MAO-I) developed as an antidepressant. It was initially designed as an antituberculosis drug. The first generation of MAO-Is (for example, tranylcypromine) consisted of irreversible, nonselective molecules with severe side effects that impeded their development.^4−6^

The second generation of MAO-Is was later marketed. They were selective but still irreversible inhibitors, typically with a propargylamine moiety (e.g., selegiline and clorgyline).^7^

Last-generation MAO-Is, which are selective and reversible, show fewer side effects. Reversible and selective MAO-A inhibitors are currently utilized as a third/fourth-line treatment for depressive disorders (e.g., moclobemide), whereas some irreversible and selective MAO-B inhibitors are used as monotherapy or as L-DOPA adjuvants (e.g., selegiline and rasagiline) in the treatment of PD.^8^

The process of creating MAO inhibitors began with the discovery of iproniazid. In this discovery process, many heterocyclic scaffolds have been used to develop new, effective derivatives.^9,10^ Moreover, pharmacophoric sites for new derivatives are clearly understood thanks to the illuminated crystal structures of both MAO isoforms of Binda.^11−13^

An amino or imino group that plays a critical role in the formation of complexes at the active site of the enzyme is a pharmacophoric group found in all inhibitors. Disubstituted hydrazines are less potent than monosubstituted analogues but can be converted to highly active monosubstituted analogues by metabolic degradation.^14,15^

Among the wide range of substituted hydrazines, several compounds act as MAO inhibitors.^14,16,17^ Cambria^18−20^ and Chimenti^21−24^ previously found that some hydrazinothiazole compounds inhibit MAO activity in the range of very low concentrations.

On the other hand, benzofuran (oxygen heterocycle) is a common structure found in a wide range of biologically active natural and medicinal compounds, and it is thus a crucial pharmacophore. It can be found in a variety of medicinally relevant molecules with biological action, such as MAO inhibitors.^25−29^

As a result of our research, we also reported the synthesis and MAO inhibitory activities of many 2-thiazolylhydrazone derivatives. This study was designed mainly to continue examining the effects of different substituents of the thiazole core at C2 and C4 on MAO inhibitory activity and selectivity.^30−33^

In this study, we introduced a benzofuran ring on the N1-hydrazine linked to the C2 of the thiazole pharmacophore through a methylene function to evaluate its impact on biological activity.

The thiazolylhydrazone ring is frequently used as an MAO inhibitor. It is supported by literature information that MAO-A activity increases when the aromatic ring is attached to the hydrazone part of the structure. In addition, it was observed that the compound containing benzofuran, one of the thiosemicarbazone derivatives synthesized by using benzofuran–benzothiophene rings, was more active. Therefore, in this study, new hybrid molecules were obtained by using benzofuran and thiazolylhydrazone pharmacophores.^18,20,22,34−39^

## 2 Results and Discussion

### 2.1 Chemistry

**Scheme 1:**
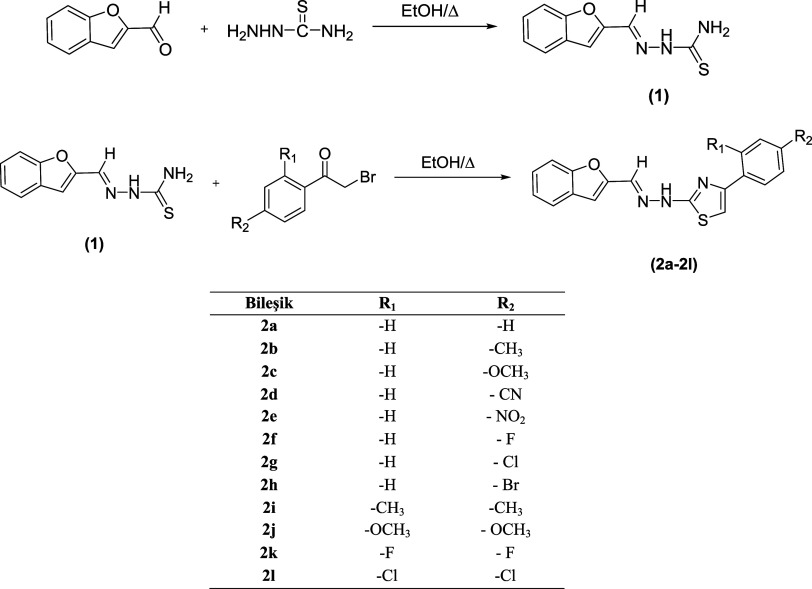
Synthesis Pathway for the Obtained Compounds (**2a**–**l**)

Designed compounds (**2a**–**l**) were obtained from two reaction steps. In the first reaction step, 2-(benzofuran-2-ylmethylene) hydrazine-1-carbothioamide (**1**) was obtained by the reaction between benzofuran-2-carbaldehyde and thiosemicarbazide in ethanol. In the second step, various phenacyl bromide derivatives were added to the solution of thiosemicarbazone (**1**) and target compounds were gained with a ring closure reaction. The synthesis scheme for obtaining the target compounds is given in Scheme 1.

### 2.2 Biological Activity

#### 2.2.1 MAO Inhibition

Inhibition profiles of the synthesized compounds on MAO enzymes were investigated by the fluorometric method. The % inhibition rates and IC_50_ values of all compounds obtained on the relevant enzymes are shown in Table 1. Biological activity results show that all of the compounds exhibit a stronger and selective inhibition profile on the MAO-A enzyme. When the inhibition results related to the MAO-B enzyme were analyzed, it was determined that all final compounds exceeded 50% inhibition rate at 10^–3^ M concentration; compounds **2a**, **2f**, **2h**, **2i**, and **2l** showed more than 50% inhibitory activity at 10^–4^ M concentrations. The IC_50_ values of these compounds on the MAO-B enzyme were calculated as 0.80 ± 0.04, 1.37 ± 0.06, 1.06 ± 0.05, 1.69 ± 0.08, and 0.75 ± 0.03 μM, respectively. Compound **2l** was determined as the compound with the strongest inhibitory activity against the MAO-B enzyme with an IC_50_ value of 0.75 ± 0.03 μM.

**Table 1:** % Inhibition Rates and IC_50_ Values of the Obtained Compounds against MAO-A and MAO-B Enzymes at 10^–3^ and 10^–4^ M Concentrations^a^

	MAO-A % inhibition			MAO-B % inhibition				
compounds	10^–3^ M	10^–4^ M	MAO-A IC_50_ (μM)	10^–3^ M	10^–4^ M	MAO-B IC_50_ (μM)	selectivity	SI*
**2a**	90.44 ± 1.66	83.66 ± 1.09	0.08 ± 0.01	85.16 ± 1.01	67.44 ± 1.29	0.80 ± 0.03	MAO-A	>9.91
**2b**	87.01 ± 1.92	78.25 ± 1.44	0.21 ± 0.01	64.51 ± 1.12	47.13 ± 0.85	>100	MAO-A	>458.72
**2c**	85.18 ± 2.03	72.76 ± 1.36	0.18 ± 0.01	55.82 ± 0.96	42.81 ± 0.89	>100	MAO-A	>568.18
**2d**	80.85 ± 1.69	70.97 ± 1.18	0.31 ± 0.01	68.99 ± 1.05	40.67 ± 0.98	>100	MAO-A	>326.79
**2e**	83.25 ± 1.65	69.32 ± 0.90	0.26 ± 0.01	72.09 ± 1.40	48.29 ± 0.86	>100	MAO-A	>386.10
**2f**	85.70 ± 1.48	73.58 ± 1.16	0.34 ± 0.01	82.73 ± 1.66	53.05 ± 1.13	1.37 ± 0.06	MAO-A	>4.06
**2g**	79.45 ± 1.06	68.67 ± 0.94	0.09 ± 0.01	68.36 ± 0.97	45.24 ± 0.86	>100	MAO-A	>1075.27
**2h**	86.80 ± 1.60	78.55 ± 1.09	0.42 ± 0.02	70.22 ± 1.36	60.92 ± 1.04	1.06 ± 0.05	MAO-A	>2.53
**2i**	84.16 ± 1.35	71.29 ± 1.86	0.43 ± 0.02	73.55 ± 1.49	57.73 ± 0.85	1.69 ± 0.08	MAO-A	>3.93
**2j**	87.50 ± 1.80	68.36 ± 1.15	0.14 ± 0.01	67.89 ± 1.30	41.55 ± 0.75	>100	MAO-A	>719.42
**2k**	89.88 ± 1.76	73.69 ± 0.83	0.13 ± 0.01	74.45 ± 1.40	40.67 ± 0.95	>100	MAO-A	>787.40
**2l**	94.00 ± 1.65	91.36 ± 1.36	0.07 ± 0.01	86.61 ± 1.29	74.21 ± 1.09	0.75 ± 0.03	MAO-A	>10.30
**Moclobemid**	94.12 ± 2.76	82.14 ± 2.69	6.06 ± 0.26				MAO-A	
**Clorgyline**	96.94 ± 1.25	91.31 ± 1.31	0.06 ± 0.01				MAO-A	
**Selegiline**				98.26 ± 1.05	96.11 ± 1.16	0.04 ± 0.01	MAO-B	

a SI*: Selectivity index (SI = IC_50_MAO-B/IC_50_ MAO-A).

When the MAO-A enzyme inhibition results were examined, all of the compounds in the series showed high activity at 10^–3^ and 10^–4^ M concentrations and showed an inhibition of more than 50%. Therefore, all compounds passed to the second stage of the MAO-A enzyme inhibition assay. Reference compounds (moclobemide, clorgyline, and selegiline) and final compounds were prepared at 10^–3^–10^–9^ M concentrations, the second-step enzyme activity was characterized, and IC_50_ values were calculated. The IC_50_ values of the reference compounds moclobemide and clorgyline were calculated as 6.06 ± 0.26 and 0.06 ± 0.01 μM, respectively. The IC_50_ values of the test compounds (**2a**–**l**) were determined in the range of 0.07 ± 0.01–0.43 ± 0.02 μM. Compound **2l** was found to be the derivative with the strongest inhibition profile against the MAO-A enzyme, with an IC_50_ value of 0.07 ± 0.01 μM. All compounds in the series were also found to show a higher inhibition capacity than that of the reference agent moclobemide on the MAO-A enzyme. Compound **2l** displayed a highly similar inhibitor profile to that of the reference drug clorgyline.

#### 2.2.2 Evaluation of Enzyme Kinetic Studies

**Figure 1:**
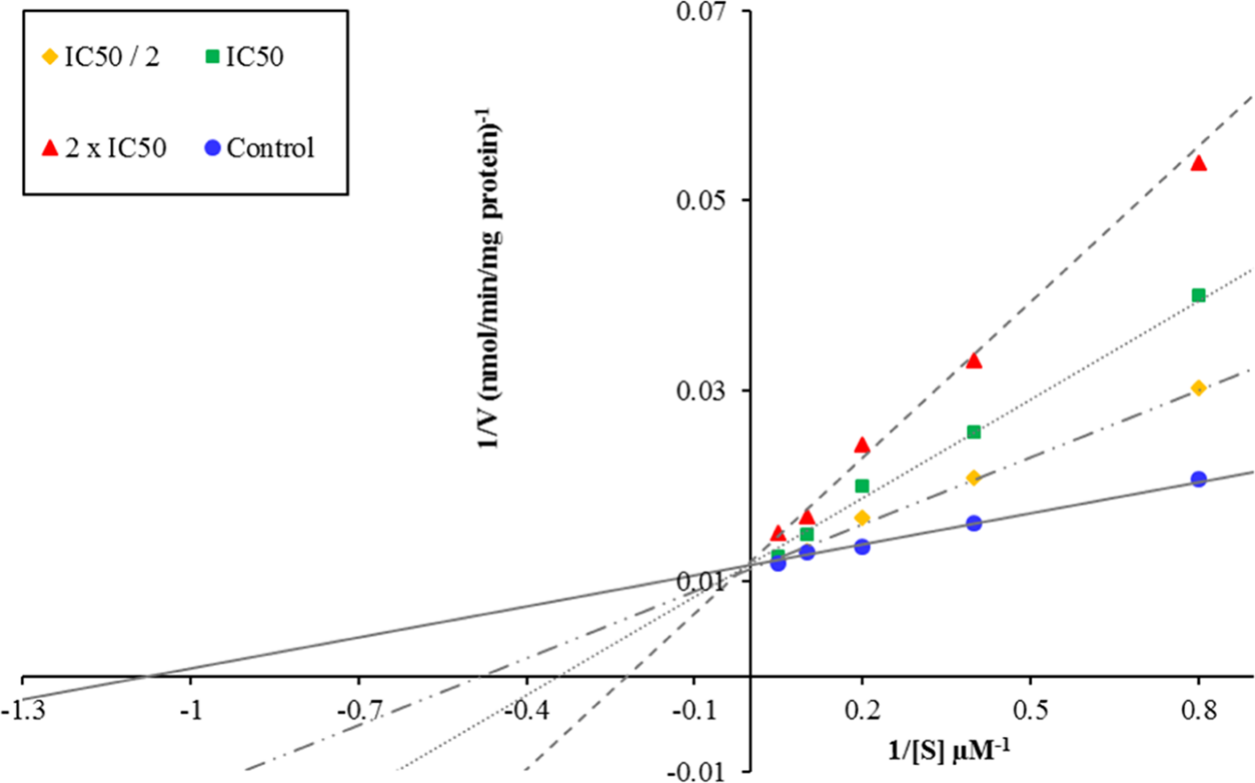
Lineweaver–Burk kinetic plot obtained by inhibition of compound **2l** by the MAO-A enzyme.

For the kinetic studies, compound **2l**, which was found to have the highest inhibitory activity on the MAO-A enzyme, was selected. Unlike enzyme activity experiments, inhibitor compounds were prepared at three different concentrations, 2 × IC_50_, IC_50_, and IC_50_/2. Substrate solutions, on the other hand, were used at six different concentrations in the range of 20–0.625 μM in MAO-A enzyme kinetic experiments (triamine). The method was applied in two different ways, in the presence and absence of an inhibitor. The Lineweaver–Burk plot was drawn by using the absorbance values and substrate concentrations obtained because of the tests. The graphs show 1/S (1/substrate concentrations) on the *x*-axis and 1/absorbance values representing 1/V on the *y*-axis (Figure 1). In the graphs, there are four different lines belonging to the concentrations of the test compounds at 2 × IC_50_, IC_50_, and IC_50_/2 values and the control group, that is, the enzyme kinetic experiment performed in the absence of an inhibitory substance. Depending on where these four lines intersect on the graph, the type of reaction between the substrate and the inhibitor against the enzyme is decided.

Enzyme inhibition is generally divided into two categories: reversible and irreversible. In irreversible inhibition, the inhibitor either covalently binds to the enzyme or forms a poorly dissociated complex. Reversible inhibition is divided into four groups: mixed type, competitive (competitive), noncompetitive (noncompetitive), and semicompetitive (uncompetitive) inhibition types. In Lineweaver–Burk plots, it is defined that if the four lines are parallel to each other, it is uncompetitive inhibition, if they intersect on the *y*-axis, it is competitive inhibition, if the intersection is on the *x*-axis, it is noncompetitive inhibition, and if there is an intersection within the regions of the graph without being on the axes, it is mixed inhibition.^39−42^

### 2.3 Blood–Brain Barrier (BBB) Permeability Prediction

**Figure 2:**
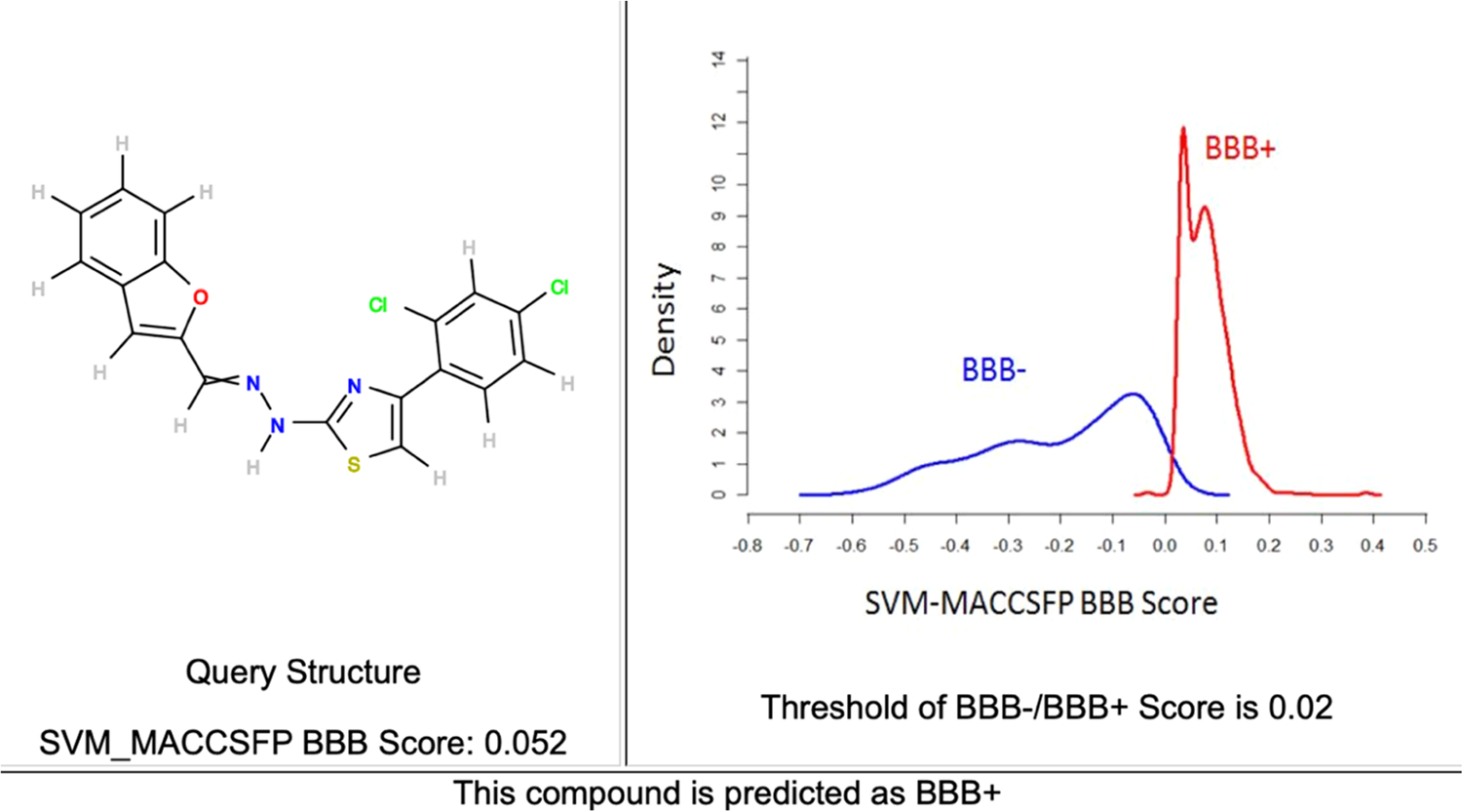
Predicted BBB permeability graphic for compound **2l**.

For drugs that act on the central nervous system to be effective, they must be able to cross the blood–brain barrier (BBB). For this purpose, the estimated BBB permeability for the active compound (compound **2l**) was calculated via an online program.^43^ The resulting graph is presented in Figure 2. Accordingly, compound **2l** is predicted to be BBB-permeable. To support the obtained in silico result, the in vitro kit method was also used and presented in the following section.

### 2.4 In Vitro BBB Permeability Assay

According to the predicted parameter, it has been observed that compound **2l** has properties that allow it to pass the BBB. To prove the accuracy of these data obtained, in vitro PAMPA tests were applied. The obtained results are presented in Table 2. Compound **2l** was found to have a high BBB permeability.

**Table 2:** Type of Blood–Brain Barrier (BBB) Penetration of Compound **2l**

classification	type of BBB permeation	compound	type of BBB permeation
**CNS+**	high BBB permeation *P*_e_ (10^–6^ cm s^–1^) > 4.0	**2l**	CNS + high BBB permeation
**CNS**–	low BBB permeation *P*_e_ (10^–6^ cm s^–1^) < 2.0		
**CNS±**	BBB permeation uncertain 2.0 < *P*_e_ (10^–6^ cm s^–1^) < 4.0		

### 2.5 Cytotoxicity Test

After the biological activity tests of the obtained compounds were completed, it was understood that the most active derivative was compound **2l**. Just as important as the effectiveness of a compound is its side effect profile. For this purpose, cytotoxic effect tests of compound **2l** were carried out. NIH3T3 (healthy mouse fibroblast cells) was used as the healthy cell in the experimental procedure. According to the obtained cytotoxic effect results, compound **2l** was found to be nontoxic with an IC_50_ value of > 100 μM.

### 2.6 Molecular Docking Study

Docking studies were carried out on the crystal structure of the MAO-A enzyme (PDB Code: 2Z5X)^44^ to determine the possible interactions of compound **2l**. This crystal structure was preferred because it was obtained from the human body (*Homo sapiens* class), its structure was clarified, and its solubility was high. In the studies, the docking technique performed with the Glide 7.1^45^ program was applied, and the grid was formed by being centered on the N5 atom of flavinin (FAD), which is in the enzyme-catalytic region.^46−48^ The most probable poses were generated with GlideScore SP.

**Figure 3:**
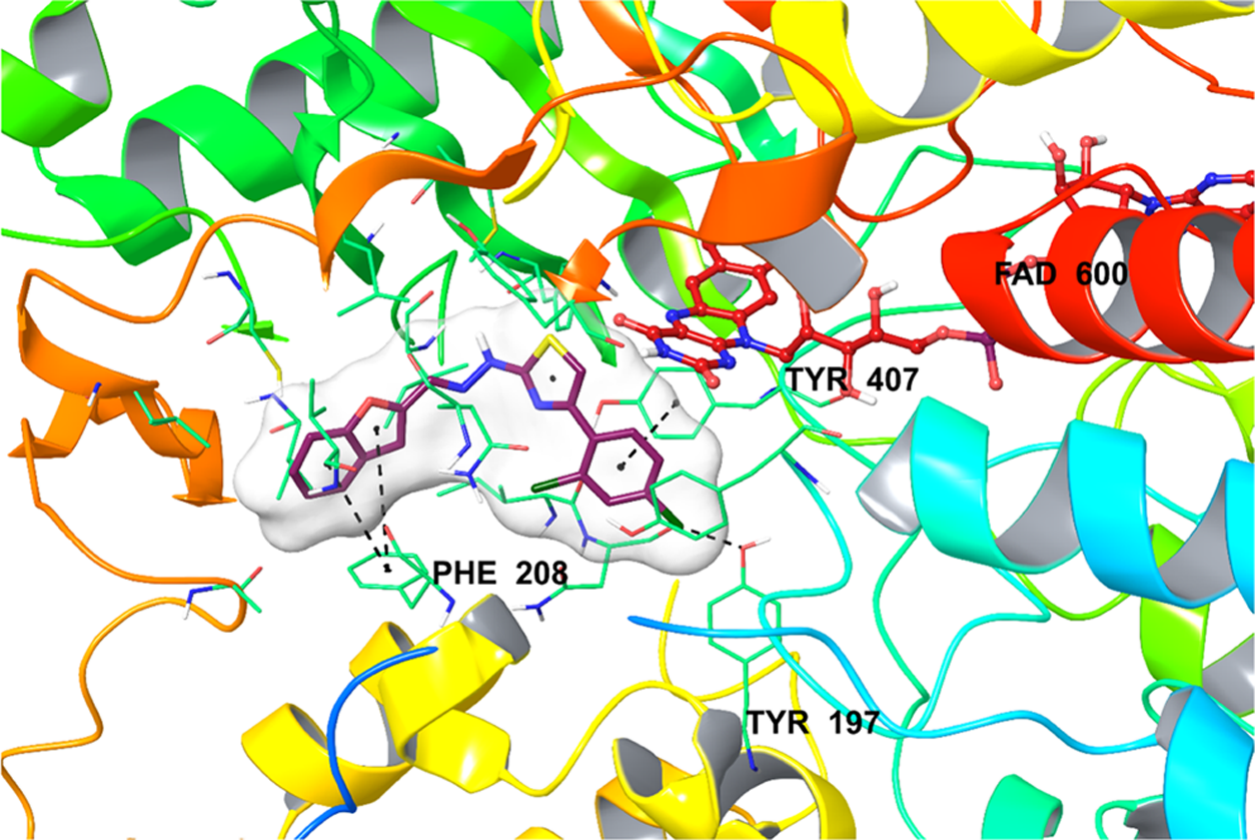
Three-dimensional view of compound **2l**’s localization and interaction at the MAO-A enzyme active site.

Looking at the docking pose of compound **2l** (Figure 3), it is seen that the benzofuran ring in the structure forms two π–π interactions with the phenyl ring of the Phe208 amino acid through both benzene and furan rings. Similarly, another π–π interaction was detected between the phenyl ring in the structure and the phenyl ring of Tyr407. In addition, it has been determined that the chlorine atom in the fourth position of this phenyl ring forms a halogen bond with the hydroxyl of amino acid Tyr197. All of these observed interactions indicate that compound **2l** binds very strongly to the MAO-A enzyme active site. These findings also explain the potent enzyme inhibitory activity of the said compound.

### 2.7 Molecular Dynamics (MD) Study

**Figure 4:**
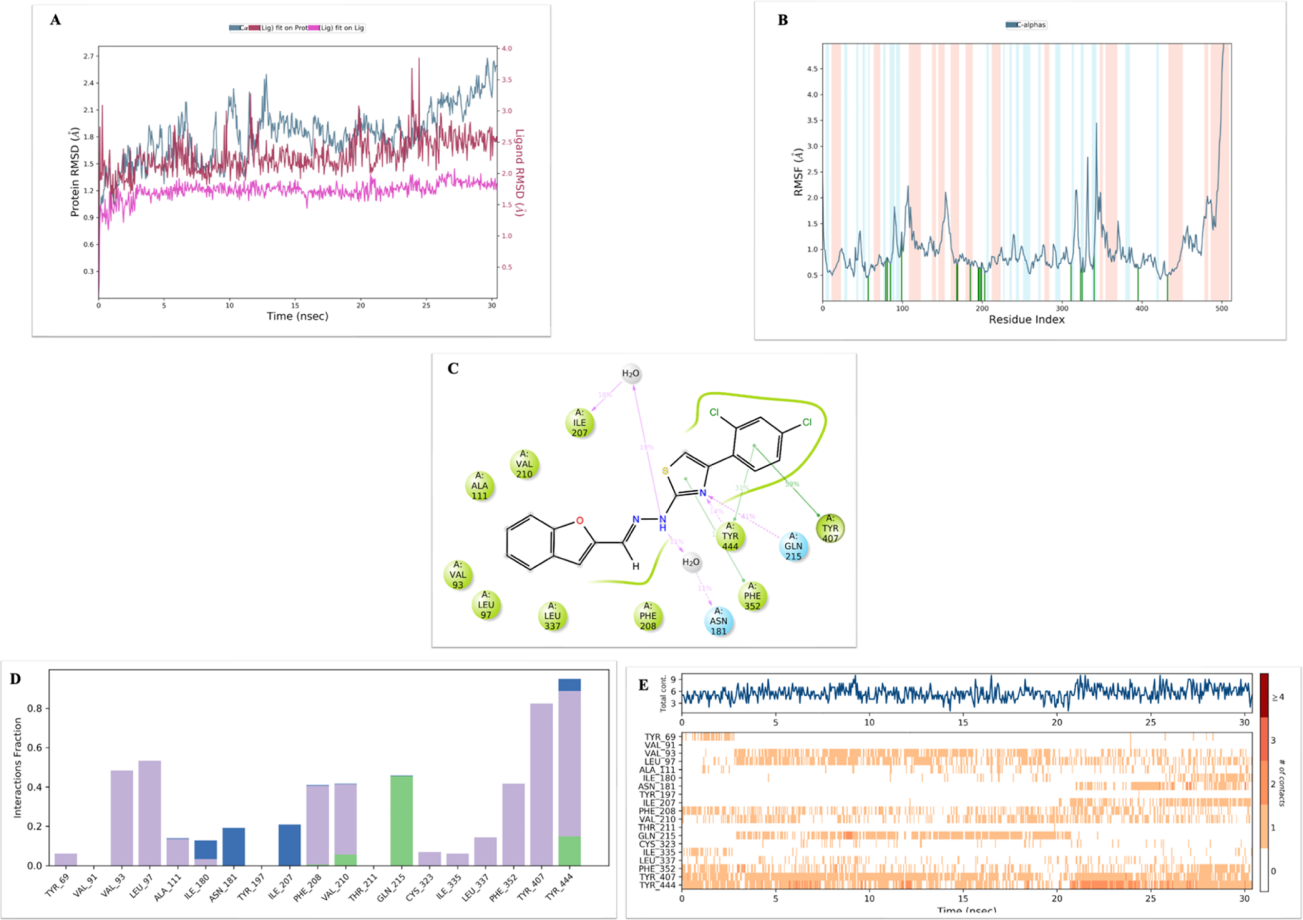
MD simulation results of complex **2l** + 2Z5X. (A) RMSD (protein RMSD is shown in gray, while the RMSD of compound **2l** is shown in red). (B) Protein RMSF. (C) 2D interaction diagram. (D) Amino acid interaction histogram. (E) Protein–ligand contact analysis of the MD trajectory.

Molecular dynamics studies were carried out to elucidate the stability and interactions of compound **2l** in the enzyme active site.^49^ The obtained reports are listed in Figure 4. Figure 4A presents root-mean-square deviation (RMSD) parameters. It is seen that the RMSD values of Cα (blue), the ligand+protein complex (red), and the ligand (pink) are portrayed. The RMSD of Cα is very important for stability. It should not exceed 3 Å. According to the obtained results, the RMSD of Cα approaches 2.7 Å maximumly as shown Figure 4A. Analyzing Figure 4B, 19 amino acid interactions are seen for RMSF parameters. We can list them in order as follows: Ala68 (0.48 Å), Lys90 (0.77 Å), Val93 (0.84 Å), Arg96 (0.75 Å), Gly110 (1.03 Å), Ile180 (0.71 Å), Asn181 (0.71 Å), Tyr197 (0.65 Å), Ile207 (0.65 Å), Phe208 (0.66 Å), Val210 (0.75 Å), Gln215 (0.53 Å), Cys323 (0.70 Å), Ser334 (1.03 Å), Leu337 (0.68 Å), Phe352 (0.92 Å), Thr407 (0.67 Å), and Thr444 (0.49 Å). Figure 4C shows a two-dimensional (2D) image of amino acids that interact with 10% or more; Figure 4D shows the relative abundance of interacting fractions; Figure 4E presents the interaction histogram over 30 ns. Uninterrupted interactions with Tyr407 and Tyr44 are particularly important here. The continuous interaction with Gln215 disappeared around 20 ns. However, this did not destabilize it. It was thought that the reason for this might be the interaction starting with Asn181 around the same ns.

In addition to these interactions, aromatic hydrogen bonds are seen in the video. While the benzofuran ring forms aromatic hydrogen bonds with Ala111, Phe177, Thr336, Cys323, and Phe208, the thiazole ring formed an aromatic hydrogen bond with Tyr407.

## 3 Conclusions

In this study, new benzofuran–thiazolylhydrazone derivatives were synthesized, and their MAO enzyme inhibitory activities were investigated. As a result of in vitro enzyme inhibition studies, 2-(2-(benzofuran-2-ylmethylene)hydrazinyl)-4-(2,4-dichlorophenyl)thiazole showed inhibitory activity against the MAO-A isoenzyme with IC_50_ = 0.073 ± 0.003 μM. The blood–brain barrier permeability of the compound was estimated physicochemically and further supported by the in vitro PAMPA assay. The fact that the compound has BBB permeability also strengthens its status as an MAO-A inhibitor candidate. The 3-(4,5-dimethylthiazol-2-yl)-2,5-diphenyl tetrazolium bromide (MTT) assay procedure was performed on NIH3T3 cells to see the cytotoxicity profile of the compound **2l**. The *in silico* studies performed for the nontoxic compound **2l** are also in agreement with the in vitro studies.

## 4 Experimental Section

### 4.1 Chemistry

All reagents were purchased from commercial suppliers and used without further purification. Melting points (mp) were determined on the Mettler Toledo-MP90 melting point system and were uncorrected. A ^1^H NMR (nuclear magnetic resonance) Bruker DPX 300 FT-NMR spectrometer and ^13^C NMR Bruker DPX 75 MHz spectrometer (Bruker Bioscience, Billerica, MA) were used. Mass spectra were recorded on an LCMS-IT-TOF (Shimadzu, Kyoto, Japan) instrument using electrospray ionization (ESI).

#### 4.1.1 Synthesis of the 2-(Benzofuran-2-ylmethylene)hydrazine-1-carbothioamide Derivative (**1**)

Benzofuran-2-carbaldehyde (5g, 0.03 mol) and thiosemicarbazide (2.73 g, 0.03 mol) were dissolved in ethanol. This mixture was refluxed for 3 h. After the end of the reaction was controlled with thin-layer chromatography (TLC), the reaction medium was cooled, and the precipitated product was filtered off. The residue was taken from the crystallization and passed to the next synthesis step.

#### 4.1.2 Synthesis of Target Compounds (**2a**–**l**)

2-(Benzofuran-2-ylmethylene)hydrazine-1-carbothioamide (**1**) (0.3 g, 0.001 mol) and substituted 2-bromoacetophenone derivatives (0.001 mol) were dissolved in ethanol. The resulting mixture was refluxed for 4 h. The end of the reaction was checked with a TLC control. The reaction vessel was cooled, and the precipitated products were removed by filtration.

##### 4.1.2.1 2-(2-(Benzofuran-2-ylmethylene)hydrazinyl)-4-phenylthiazole (**2a**)

Yield: 80%, ^1^H NMR (300 MHz, DMSO-*d*_6_): δ = 7.24–7.35 (8H, m, Ar–H), 7.56–7.69 (3H, m, Ar–H), 8.06 (1H, s, –CH = N–). ^13^C NMR (75 MHz, DMSO-*d*_6_): δ = 105.23, 109.56, 111.82, 122.11, 123.92, 126.24, 128.32, 128.39, 128.98, 132.48, 134.48, 147.88, 151.49, 155.07, 158.83, 166.31. HRMS (mz): [M + H]^+^ calculated C_18_H_13_N_3_OS: 320.0852; found: 320.0863.

##### 4.1.2.2 2-(2-(Benzofuran-2-ylmethylene)hydrazinyl)-4-(*p*-tolyl)thiazole (**2b**)

Yield: 83%, ^1^H NMR (300 MHz, DMSO-*d*_6_): δ = 2.35 (3H, s, –CH_3_), 7.23–7.29 (5H, m, Ar–H), 7.34–7.39 (1H, m, Ar–H), 7.64–7.67 (2H, m, Ar–H), 7.74–7.78 (2H, m, Ar–H), 8.06 (1H, s, –CH=N–). ^13^C NMR (75 MHz, DMSO-*d*_6_): δ = 21.28, 103.71, 111.81, 122.18, 123.91, 125.96, 128.23, 129.38, 129.67, 131.16, 131.65, 132.30, 132.66, 137.39, 138.23, 155.05, 166.79. HRMS (mz): [M + H]^+^ calculated: C_19_H_15_N_3_OS: 334.1009; found: 334.1020.

##### 4.1.2.3 2-(2-(Benzofuran-2-ylmethylene)hydrazinyl)-4-(4-methoxyphenyl)thiazole (**2c**)

Yield: 77%, ^1^H NMR (300 MHz, DMSO-*d*_6_): δ = 3.74 (3H, s, -OCH_3_), 6.82 (2H, d, *J* = 8.9 Hz, Ar–H), 6.94–6.99 (1H, m, Ar–H), 7.25–7.29 (5H, m, Ar–H), 7.62–7.66 (2H, m, Ar–H), 8.06 (1H, s, –CH=N–). ^13^C NMR (75 MHz, DMSO-*d*_6_): δ = 55.54, 105.13, 109.40, 111.89, 114.33, 122.05, 123.88, 126.14, 127.33, 128.29, 128.43, 129.69, 151.59, 155.09, 159.04, 159.45, 166.14. HRMS (mz): [M + H]^+^ calculated: C_19_H_15_N_3_O_2_S: 350.0958; found: 350.0969.

##### 4.1.2.4 4-(2-(2-(Benzofuran-2-ylmethylene)hydrazinyl)thiazol-4-yl)benzonitrile (**2d**)

Yield: 79%, ^1^H NMR (300 MHz, DMSO-*d*_6_): δ = 7.25–7.26 (1H, m, Ar–H), 7.27–7.30 (1H, m, Ar–H), 7.38 (1H, td, *J*_1_ = 1.3 Hz, *J*_2_ = 7.3 Hz, Ar–H), 7.64–7.69 (3H, m, Ar–H), 7.88 (2H, d, *J* = 8.5 Hz, Ar–H), 8.03 (1H, s, –CH=N–), 8.06–8.08 (2H, m, Ar–H), 12.49 (1H, s, –NH). ^13^C NMR (75 MHz, DMSO-*d*_6_): δ = 108.46, 109.28, 110.14, 111.80, 119.44, 122.11, 123.93, 126.26, 126.60, 128.44, 132.09, 133.20, 139.11, 149.34, 151.61, 155.09, 168.44. HRMS (mz): [M + H]^+^ calculated: C_19_H_12_N_4_OS: 345.0805; found: 345.0812.

##### 4.1.2.5 2-(2-(Benzofuran-2-ylmethylene)hydrazinyl)-4-(4-nitrophenyl)thiazole (**2e**)

Yield: 85%, ^1^H NMR (300 MHz, DMSO-*d*_6_): δ = 7.25–7.28 (1H, m, Ar–H), 7.27–7.30 (1H, m, Ar–H), 7.38 (1H, td, *J*_1_ = 1.3 Hz, *J*_2_ = 7.3 Hz, Ar–H), 7.65–7.69 (2H, m, Ar–H), 7.77 (1H, s, Ar–H), 8.08–8.13 (3H, m, Ar–H), 8.29 (2H, d, *J* = 9.0 Hz, Ar–H), 12.53 (1H, s, –NH). ^13^C NMR (75 MHz, DMSO-*d*_6_): δ = 109.37, 109.47, 111.81, 122.13, 123.93, 124.62, 126.28, 126.84, 128.43, 132.16, 141.00, 146.74, 149.03, 151.58, 155.10, 168.55. HRMS (mz): [M + H]^+^ calculated: C_18_H_12_N_4_O_3_S: 365.0703; found: 365.0711.

##### 4.1.2.6 2-(2-(Benzofuran-2-ylmethylene)hydrazinyl)-4-(4-fluorophenyl)thiazole (**2f**)

Yield: 77%, ^1^H NMR (300 MHz, DMSO-*d*_6_): δ = 7.06–7.13 (2H, m, Ar–H), 7.24–7.37 (5H, m, Ar–H), 7.62–7.66 (3H, m, Ar–H), 8.06 (1H, s, –CH=N–). ^13^C NMR (75 MHz, DMSO-*d*_6_): δ = 105.39, 109.58, 111.88, 115.87 (d, *J* = 21.4 Hz), 122.09, 123.89, 126.21, 128.40, 130.47 (d, *J* = 8.1 Hz), 131.02, 132.49, 151.49, 155.09, 158.42, 162.16 (d, *J* = 244.3 Hz), 166.35. HRMS (mz): [M + H]^+^ calculated: C_18_H_12_N_3_OFS: 338.0758; found: 338.0774.

##### 4.1.2.7 2-(2-(Benzofuran-2-ylmethylene)hydrazinyl)-4-(4-chlorophenyl)thiazole (**2g**)

Yield: 81%, ^1^H NMR (300 MHz, DMSO-*d*_6_): δ = 7.24–7.32 (7H, m, Ar–H), 7.63–7.69 (3H, m, Ar–H), 8.06 (1H, s, –CH=N–). ^13^C NMR (75 MHz, DMSO-*d*_6_): δ = 105.47, 109.64, 111.89, 122.09, 123.89, 125.26, 126.22, 127.71, 128.40, 128.99, 130.09, 133.27, 151.47, 155.11, 158.16, 166.46. HRMS (mz): [M + H]^+^ calculated: C_18_H_12_N_3_OSCl: 354.0462; found: 354.0473.

##### 4.1.2.8 2-(2-(Benzofuran-2-ylmethylene)hydrazinyl)-4-(4-bromophenyl)thiazole (**2h**)

Yield: 81%, ^1^H NMR (300 MHz, DMSO-*d*_6_): δ = 7.24–7.30 (5H, m, Ar–H), 7.44–7.47 (2H, m, Ar–H), 7.62–7.66 (3H, m, Ar–H), 8.06 (1H, s, –CH=N–). ^13^C NMR (75 MHz, DMSO-*d*_6_): δ = 105.48, 109.12, 109.66, 111.89, 121.92, 123.89, 126.21, 128.03, 128.40, 130.37, 131.93, 133.71, 151.47, 155.11, 158.12, 166.50. HRMS (mz): [M + H]^+^ calculated: C_18_H_12_N_3_OSBr: 397.9957; found: 397.9975.

##### 4.1.2.9 2-(2-(Benzofuran-2-ylmethylene)hydrazinyl)-4-(2,4-dimethylphenyl)thiazole (**2i**)

Yield: 75%, ^1^H NMR (300 MHz, DMSO-*d*_6_): δ = 2.29 (3H, s, –CH_3_), 2.42 (3H, s, –CH_3_), 6.91 (1H, s, Ar–H), 7.02–7.07 (2H, m, Ar–H), 7.22–7.24 (1H, m, Ar–H), 7.27–7.30 (1H, m, Ar–H), 7.36–7.39 (1H, m, Ar–H), 7.49 (1H, d, *J* = 7.7 Hz, Ar–H), 7.65–7.68 (2H, m, Ar–H), 8.06 (1H, s, –CH=N–), 12.33 (1H, s, –NH). ^13^C NMR (75 MHz, DMSO-*d*_6_): δ = 21.11, 21.56, 106.65, 108.76, 111.77, 122.01, 123.88, 126.09, 126.87, 128.51, 129.61, 131.47, 131.90, 132.31, 135.54, 137.20, 151.07, 151.87, 155.05, 167.14. HRMS (mz): [M + H]^+^ calculated: C_20_H_17_N_3_OS: 348.1165; found: 348.1175.

##### 4.1.2.10 2-(2-(Benzofuran-2-ylmethylene)hydrazinyl)-4-(2,4-dimethoxyphenyl)thiazole (**2j**)

Yield: 75%, ^1^H NMR (300 MHz, DMSO-*d*_6_): δ = 3.81 (3H, s, -OCH_3_), 3.91 (3H, s, -OCH_3_), 6.58–6.66 (2H, m, Ar–H), 7.24–7.28 (3H, m, Ar–H), 7.34–7.38 (1H, m, Ar–H), 7.64–7.67 (2H, m, Ar–H), 7.93 (1H, d, *J* = 8.5 Hz, Ar–H), 8.03–8.06 (1H, m, –CH=N–). ^13^C NMR (75 MHz, DMSO-*d*_6_): δ = 55.79, 56.01, 99.11, 105.50, 108.91, 109.44, 111.78, 122.05, 122.12, 123.92, 123.99, 126.14, 126.30, 128.44, 130.21, 131.63, 132.16, 155.09, 160.33, 166.41. HRMS (mz): [M + H]^+^ calculated C_20_H_17_N_3_O_3_S: 380.1063; found: 380.1070.

##### 4.1.2.11 2-(2-(Benzofuran-2-ylmethylene)hydrazinyl)-4-(2,4-difluorophenyl)thiazole (**2k**)

Yield: 75%, ^1^H NMR (300 MHz, DMSO-*d*_6_): δ = 6.96–7.02 (1H, m, Ar–H), 7.10–7.17 (1H, m, Ar–H), 7.23–7.35 (5H, m, Ar–H), 7.64–7.68 (2H, m, Ar–H), 8.04 (1H, s, –CH=N–), 12.46 (1H, s, –NH). ^13^C NMR (75 MHz, DMSO-*d*_6_): δ = 109.19, 110.14, 111.80, 122.09, 123.92, 126.22, 128.00, 128.45, 130.26, 131.88, 132.06, 132.51, 132.68, 133.05, 146.43, 151.66, 155.08, 167.37. HRMS (mz): [M + H]^+^ calculated: C_18_H_11_N_3_OF_2_S: 356.0664; found: 356.0679.

##### 4.1.2.12 2-(2-(Benzofuran-2-ylmethylene)hydrazinyl)-4-(2,4-dichlorophenyl)thiazole (**2l**)

Yield: 75%, ^1^H NMR (300 MHz, DMSO-*d*_6_): δ = 7.25 (1H, s, Ar–H), 7.27–7.30 (1H, m, Ar–H), 7.34–7.39 (1H, m, Ar–H), 7.44 (1H, s, Ar–H), 7.49–7.54 (1H, m, Ar–H), 7.65–7.70 (3H, m, Ar–H), 7.89 (1H, d, *J* = 8.5 Hz, Ar–H), 8.06 (1H, s, –CH=N–), 12.45 (1H, s, –NH). ^13^C NMR (75 MHz, DMSO-*d*_6_): δ = 109.19, 110.14, 111.80, 122.09, 123.92, 126.22, 128.00, 128.45, 130.26, 131.88, 132.06, 132.51, 132.68, 133.05, 146.43, 151.66, 155.08, 167.37. HRMS (mz): [M + H]^+^ calculated: C_18_H_11_N_3_OSCl_2_: 388.0073; found: 388.0081.

### 4.2 Activity Studies

#### 4.2.1 MAO-A and MAO-B Enzyme Inhibition Study

In every step of the method, distilled water obtained from a Millipor, a Milli-Q Synthesis A10 purification device, was used. Care was taken in preparing all of the solutions used fresh and to consume them within 1 week after preparation. A BioTek-Precision Power robotic pipetting system was used in the processes of separating the solutions prepared in the enzyme inhibition study, applying the test compounds to 96-well plates, and adding the enzyme–substrate solutions. The creation, monitoring, and spectrophotometric measurements of the enzyme protocol were performed on a BioTek-Synergy H1 microplate reader.^41,42^ The IC_50_ values of the selected compounds and standard agent were determined by nonlinear regression analysis over the calculated % inhibition values at the concentrations between 10^–3^ and 10^–9^ M with the help of GraphPad Prism Version 6 software since these compounds showed more selectivity toward MAO-A than for MAO-B.

### 4.3 BBB Permeability Prediction

The estimated BBB permeability for the active compound (compound **2l**) was calculated via an online program.^43^

### 4.4 In Vitro BBB Permeability Assay

To observe the BBB crossing ability of the most active compound **2l**, the parallel artificial membrane permeability assay (PAMPA) was performed as previously described.^50,51^

#### 4.4.1 Cytotoxicity Tests

3-(4,5-Dimethylthiazol-2-yl)-2,5-diphenyl tetrazolium bromide (MTT) stock solution (5 mg mL^–1^) was prepared by dissolving in phosphate buffer (PBS). The viable cell count of NIH/3T3 (mouse healthy embryo fibroblast cells) cells grown in appropriate medium and culture medium was characterized, and each cell line was seeded in 96-well plates with 3000 cells in each well, and the cells were incubated for 24 h for their adhesion. The old medium in the plates was discarded, and the concentrations of the compounds prepared freshly in the culture medium were applied to the wells. Medium containing 0.1% dimethyl sulfoxide (DMSO) was applied to the cells in the control group. The plates were then allowed to incubate for 24 h. At the end of the incubation period, the medium in the plate was discarded and 100 μL of MTT working solution (final concentration of 0.5 mg mL^–1^) was added to the cells in each of the 96 wells, and the cells were incubated in the incubator for 3 h. At the end of the incubation, the medium in each well was removed and 100 μL of DMSO was added as a solvent and the absorbance values were read in an enzyme-linked immunosorbent assay (ELISA) device (Bio Tek Cytation3 multimode microplate reader) at a wavelength of 540 nm, with eight wells in each group. Experiments are run as three independent repetitions. The obtained absorbance values give the metabolic activities of the cells, and these values are associated with the number of living cells. The results are determined as % inhibition values according to the formula given, and IC_50_ values are calculated from these values by using the Excel program.^52−54^

### 4.5 Molecular Docking

Molecular docking studies were performed using an in silico procedure to define the binding modes of the active compound (**2l**) in the active regions of crystal structures of MAO-A (PDB ID: 2Z5X),^44^ retrieved from the Protein Data Bank server (www.pdb.org, accessed 01 May 2021). Molecular docking studies were performed as previously reported.^31,39,45,55,56^

### 4.6 Molecular Dynamics Simulation

Molecular dynamics (MD) simulations, which are considered an important computational tool to evaluate the time-dependent stability of a ligand at an active site for a drug–receptor complex, were performed for compound **2l** within the scope of this study.^49^ Molecular dynamics studies were performed for 30 ns as previously reported.^57,58^

## Supporting Information Available

^1^H NMR, ^13^C NMR, and HRMS spectra of compounds **2a**–**l** (PDF)
