# Supplementary material for: Design, Synthesis, and Biological Effect Studies of Novel Benzofuran–Thiazolylhydrazone Derivatives as Monoamine Oxidase Inhibitors
Source: ACS Omega. 2024 Feb 29;9(10):11388–97. doi: 10.1021/acsomega.3c07703 (PMC10938434; doi:10.1021/acsomega.3c07703)
Supplement: Supplementary file 1 — ao3c07703_si_001.pdf [file ao3c07703_si_001.pdf]

Design, Synthesis and Biological Effects Studies of Novel Benzofuran-Thiazolylhydrazone Derivatives as Monoamine Oxidase Inhibitors

Derya OSMANİYE [a,b*], Begüm Nurpelin SAĞLIK [a,b], Serkan LEVENT [a, b] , Ulviye ACAR ÇEVİK [a,b], Sinem ILGIN [c], Leyla YURTTAŞ [a], Yusuf ÖZKAY [a,b], Ahmet Cagri KARABURUN [a], Zafer Asım KAPLANCIKLI [a], Nalan GUNDOGDU-KARABURUN [a*]

[a] *Department of Pharmaceutical Chemistry, Faculty of Pharmacy, Anadolu University, 26470 Eskişehir, Turkey*

[b] *Central Research Laboratory (MERLAB), Faculty of Pharmacy, Anadolu University, 26470 Eskişehir, Turkey*

[c] *Department of Pharmaceutical Toxicology, Faculty of Pharmacy, Anadolu University, 26470 Eskişehir, Turkey*

* Corresponding author.

E-mail address: ngundogd@anadolu.edu.tr (N. Gundogdu-Karaburun); dosmaniye@anadolu.edu.tr (D. Osmaniye)

Tel: +90-222-3350580/3778 *Fax*: +90-222-3350750.

Address: Anadolu University, Faculty of Pharmacy, Department of Pharmaceutical Chemistry, 26470, Eskişehir, Turkey.

Data File: C:\LabSolutions\Data\Analiz\derya\KD-2a_19.lcd

Elmt	Val.	Min	Max	Elmt	Val.	Min	Max	Elmt	Val.	Min	Max	Elmt	Val.	Min	Max	Use Adduct
H	1	6	40	O	2	0	5	S	2	1	1	Ru	2	0	0	H
C	4	7	35	F	1	0	0	Cl	1	0	0	Pd	2	0	0	Na
N	3	3	5	P	3	0	0	Br	1	0	0	I	3	0	0	

Error Margin (ppm):	5	DBE Range:	10.0 - 25.0	Electron Ions:	both
HC Ratio:	unlimited	Apply N Rule:	yes	Use MSn Info:	yes
Max Isotopes:	3	Isotope RI (%):	1.00	Isotope Res:	9000
MSn Iso RI (%):	10.00	MSn Logic Mode:	AND	Max Results:	100

Event#: 1 MS(E+) Ret. Time : 1.840 Scan# : 277

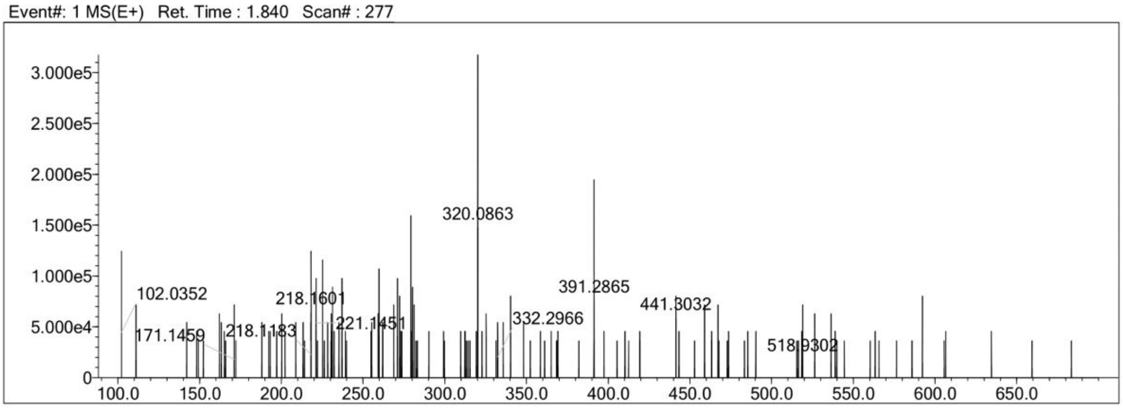

Measured region for 320.0863 m/z

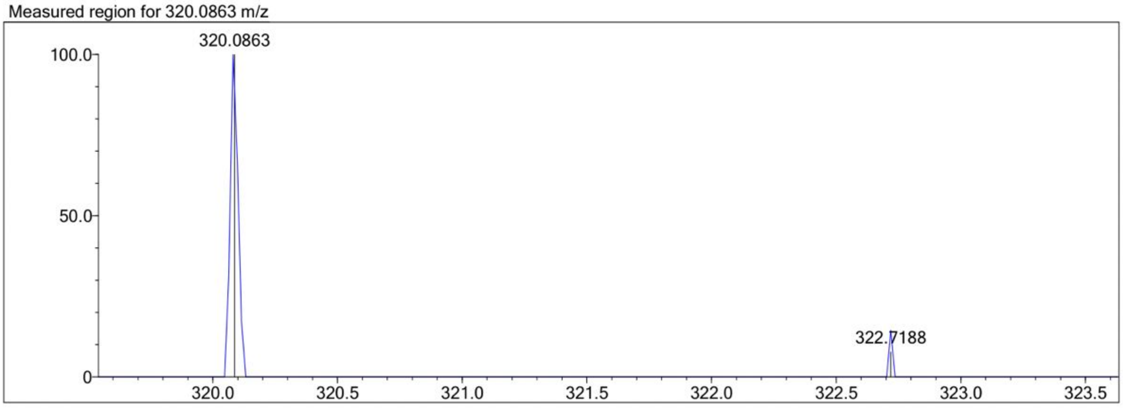

C18 H13 N3 O S [M+H]+ : Predicted region for 320.0852 m/z

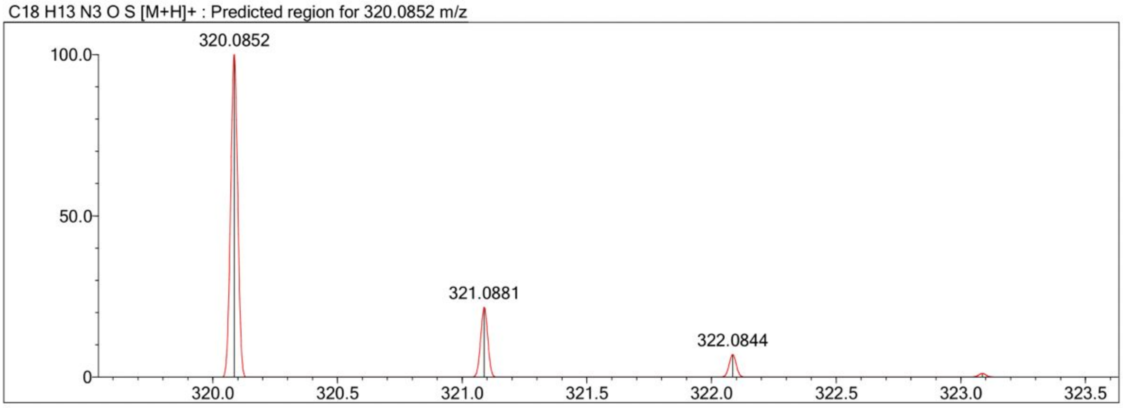

Rank	Score	Formula (M)	Ion	Meas. m/z	Pred. m/z	Df. (mDa)	Df. (ppm)	Iso	DBE
1	0.00	C18 H13 N3 O S	[M+H]+	320.0863	320.0852	1.1	3.44	0.00	14.0

Figure S1. HRMS spectra of compound **2a**

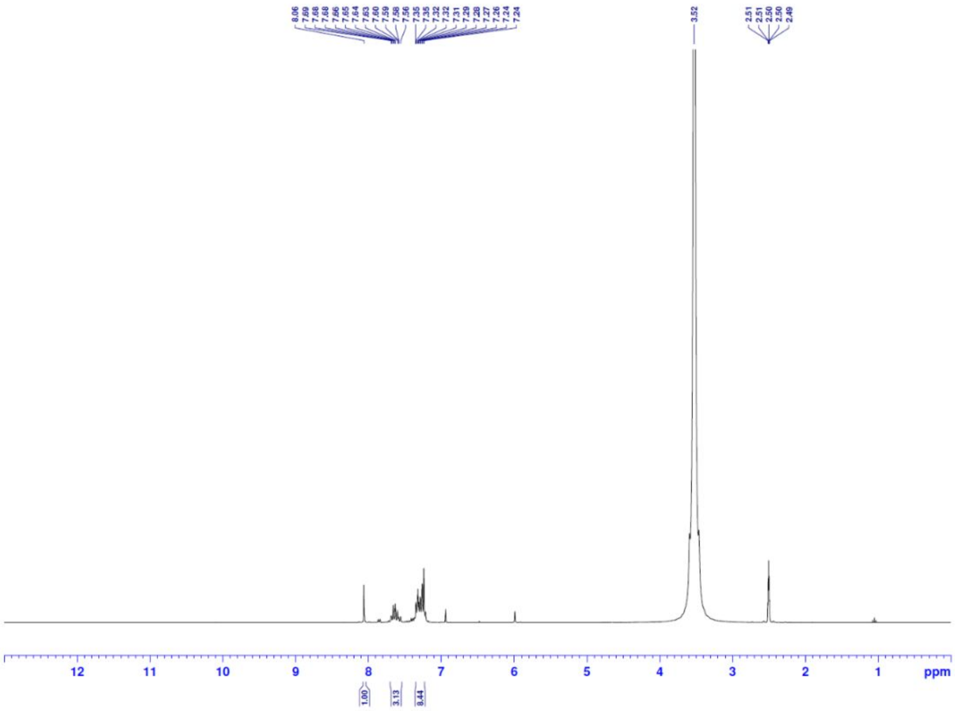

Figure S2. ^{1}H-NMR spectra of compound **2a**

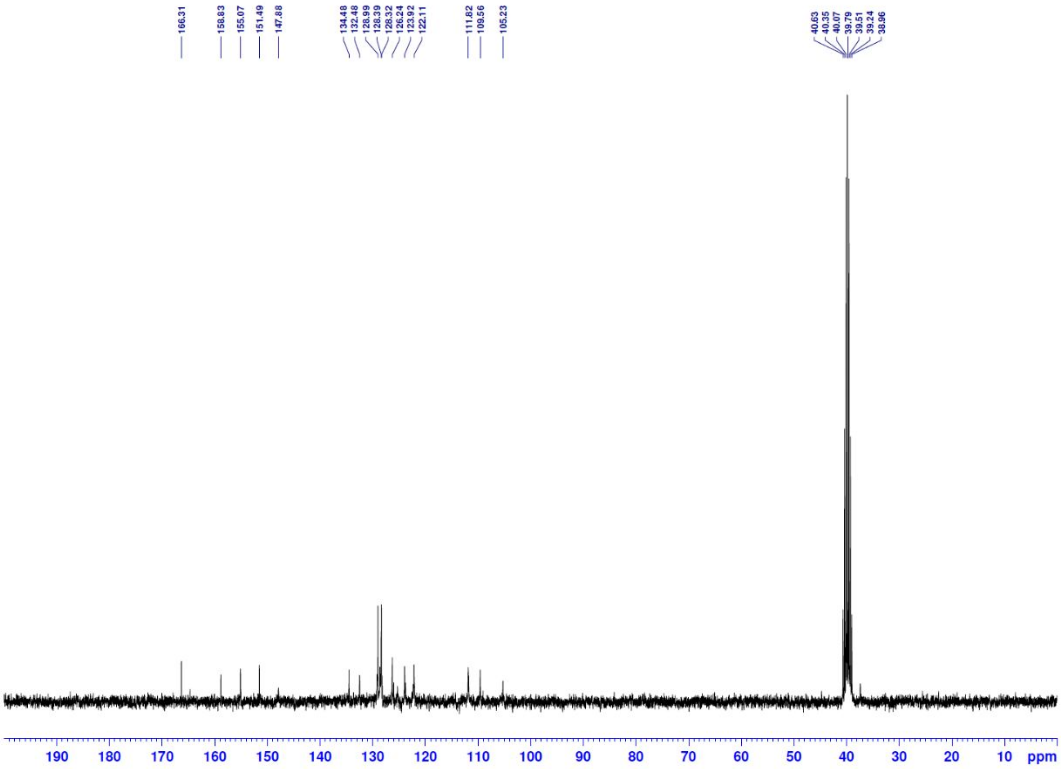

Figure S3. ^{13}C-NMR spectra of compound **2a**

Data File: C:\LabSolutions\Data\Analiz\derya\KD-2b_20.lcd

Elmt	Val.	Min	Max	Elmt	Val.	Min	Max	Elmt	Val.	Min	Max	Elmt	Val.	Min	Max	Use Adduct
H	1	6	40	O	2	0	3	S	2	1	1	Ru	2	0	0	H
C	4	7	35	F	1	0	0	Cl	1	0	1	Pd	2	0	0	Na
N	3	0	4	P	3	0	0	Br	1	0	0	I	3	0	0	

Error Margin (ppm):	5	DBE Range:	10.0 - 25.0	Electron Ions:	both
HC Ratio:	unlimited	Apply N Rule:	yes	Use MSn Info:	yes
Max Isotopes:	3	Isotope RI (%):	1.00	Isotope Res:	9000
MSn Iso RI (%):	10.00	MSn Logic Mode:	AND	Max Results:	100

Event#: 1 MS(E+) Ret. Time : 2.187 Scan# : 329

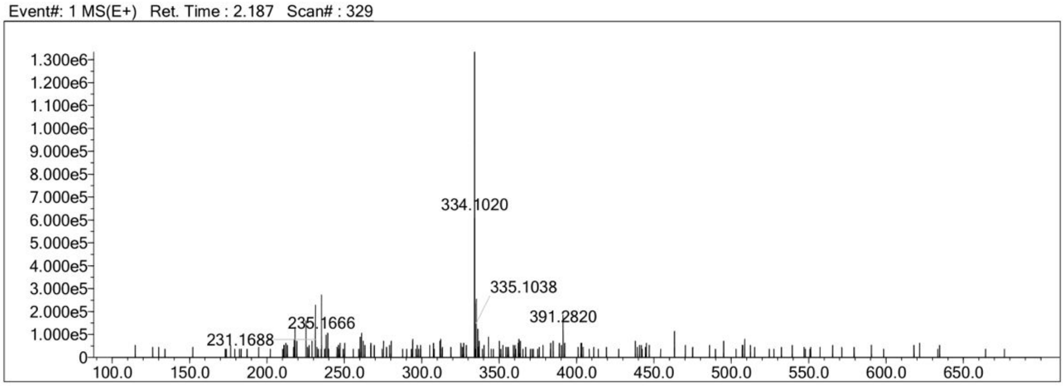

Measured region for 334.1020 m/z

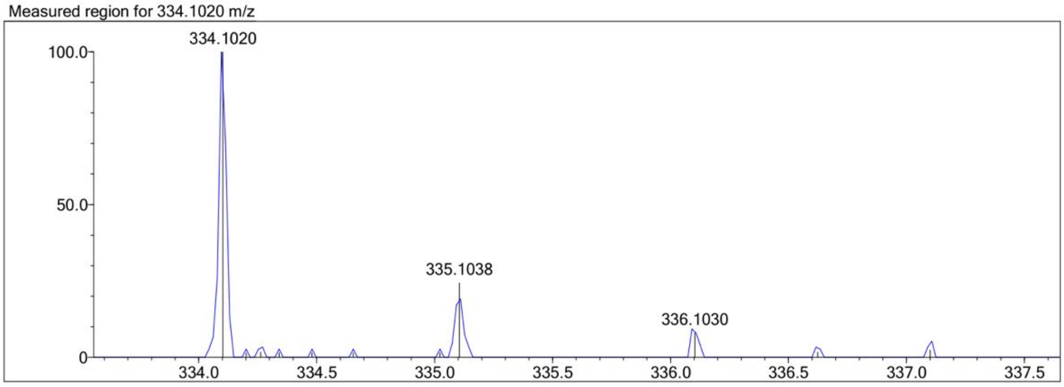

C19 H15 N3 O S [M+H]+ : Predicted region for 334.1009 m/z

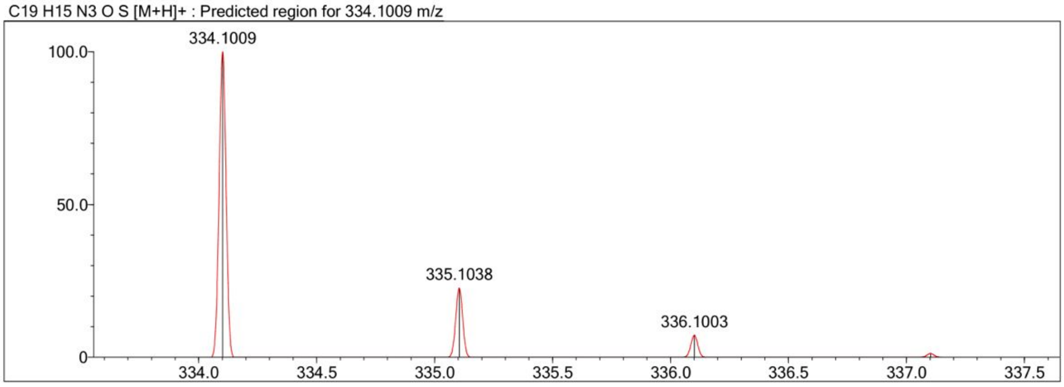

Rank	Score	Formula (M)	Ion	Meas. m/z	Pred. m/z	Df. (mDa)	Df. (ppm)	Iso	DBE
1	61.84	C19 H15 N3 O S	[M+H]+	334.1020	334.1009	1.1	3.29	65.59	14.0

Figure S4. HRMS spectra of compound **2b**

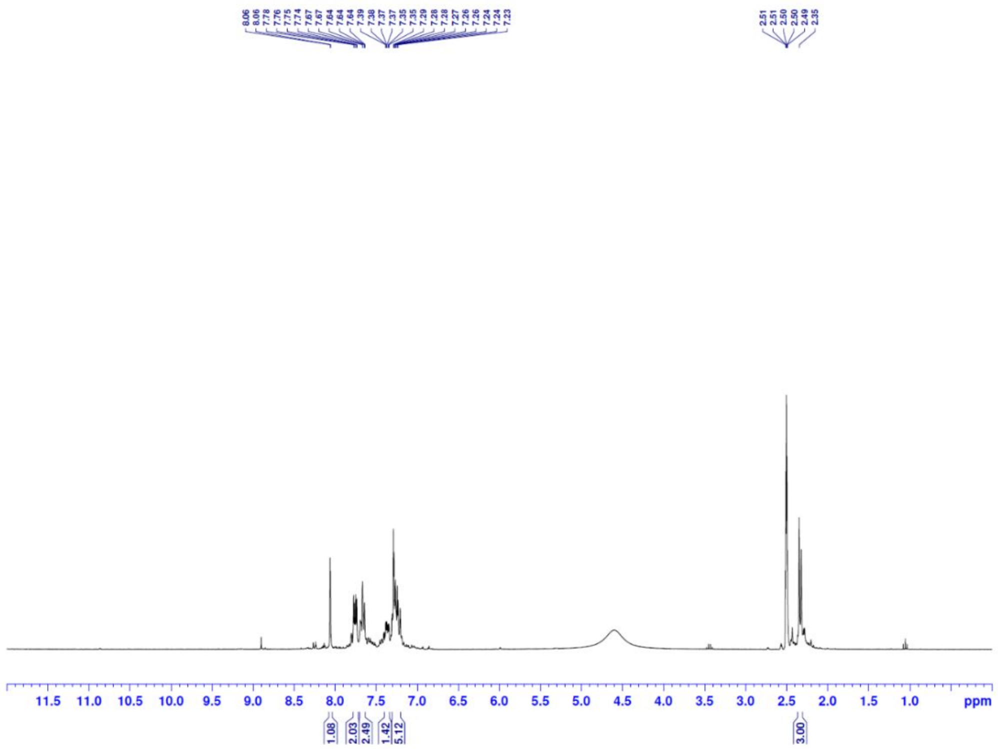

Figure S5. ^{1}H-NMR spectra of compound **2b**

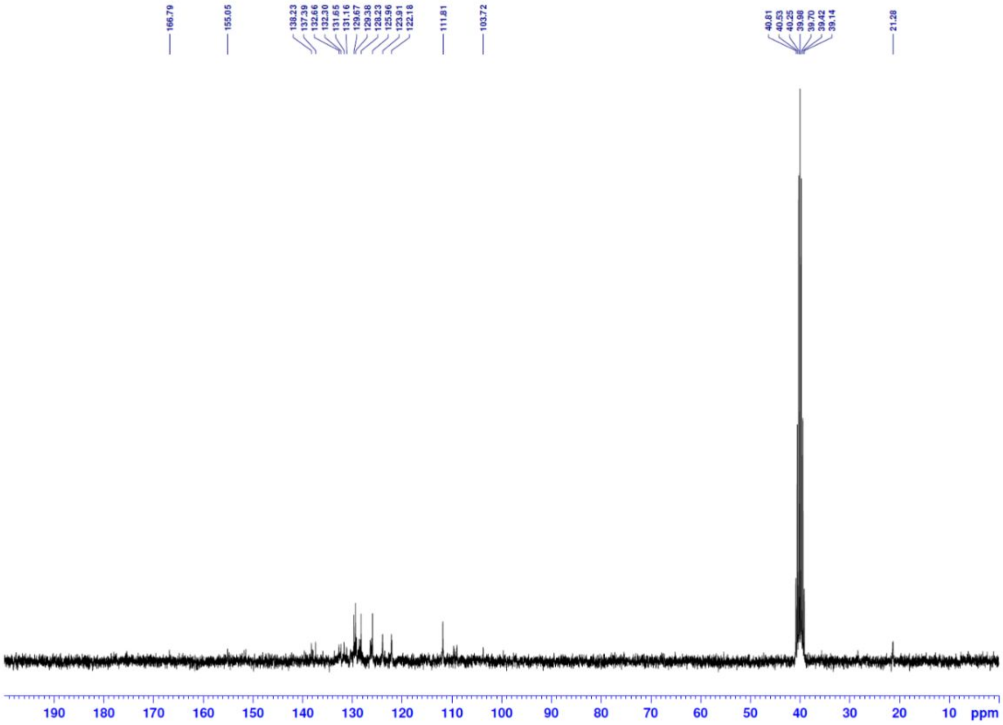

Figure S6. ^{13}C-NMR spectra of compound **2b**

Formula Predictor Report - KD-2c_21.lcd

Data File: C:\LabSolutions\Data\Analiz\derya\KD-2c_21.lcd

Elmt	Val.	Min	Max	Elmt	Val.	Min	Max	Elmt	Val.	Min	Max	Elmt	Val.	Min	Max	Use Adduct
H	1	6	40	O	2	0	5	S	2	1	1	Ru	2	0	0	H
C	4	7	35	F	1	0	0	Cl	1	0	0	Pd	2	0	0	Na
N	3	3	5	P	3	0	0	Br	1	0	0	I	3	0	0	

Error Margin (ppm):	5	DBE Range:	10.0 - 25.0	Electron Ions:	both
HC Ratio:	unlimited	Apply N Rule:	yes	Use MSn Info:	yes
Max Isotopes:	3	Isotope RI (%):	1.00	Isotope Res:	9000
MSn Iso RI (%):	10.00	MSn Logic Mode:	AND	Max Results:	100

Event#: 1 MS(E+) Ret. Time : 2.240 Scan# : 337

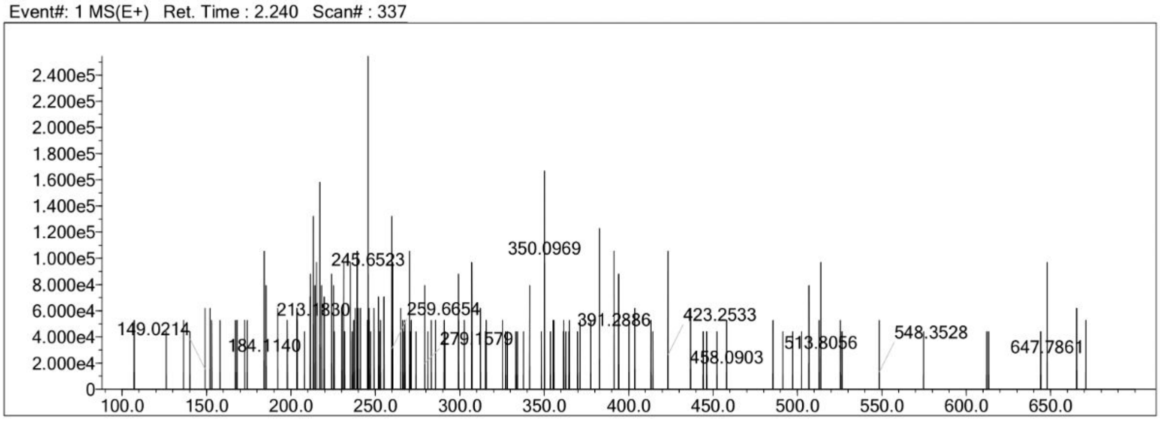

Measured region for 350.0969 m/z

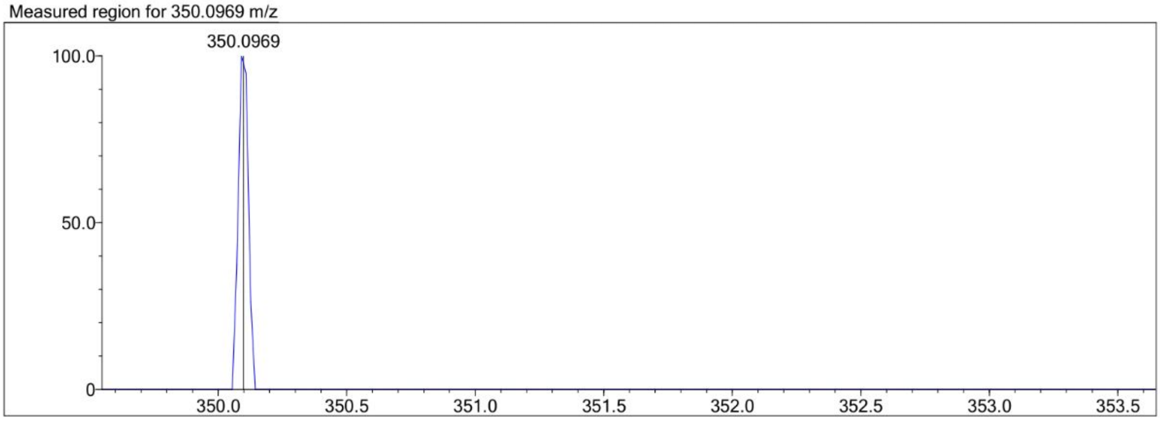

C19 H15 N3 O2 S [M+H]+ : Predicted region for 350.0958 m/z

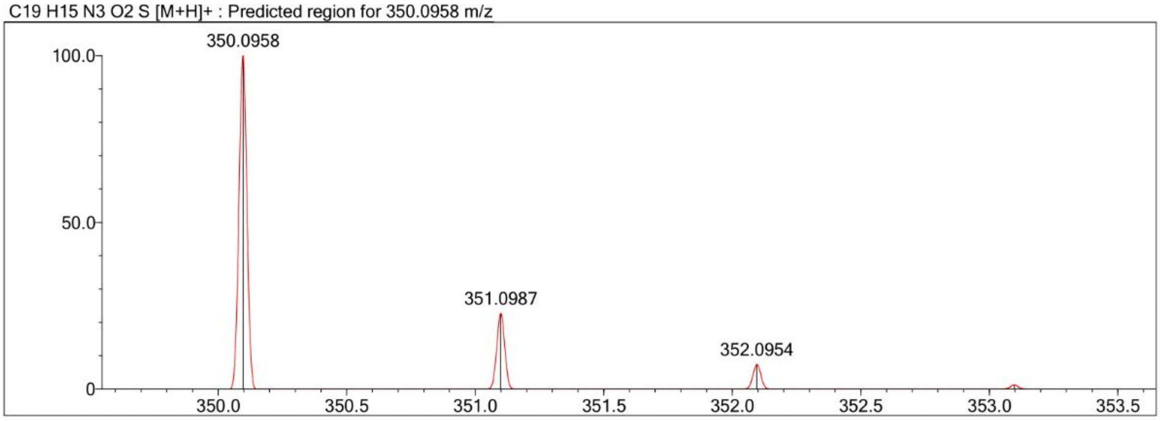

Rank	Score	Formula (M)	Ion	Meas. m/z	Pred. m/z	Df. (mDa)	Df. (ppm)	Iso	DBE
1	0.00	C19 H15 N3 O2 S	[M+H]+	350.0969	350.0958	1.1	3.14	0.00	14.0

Figure S7. HRMS spectra of compound **2c**

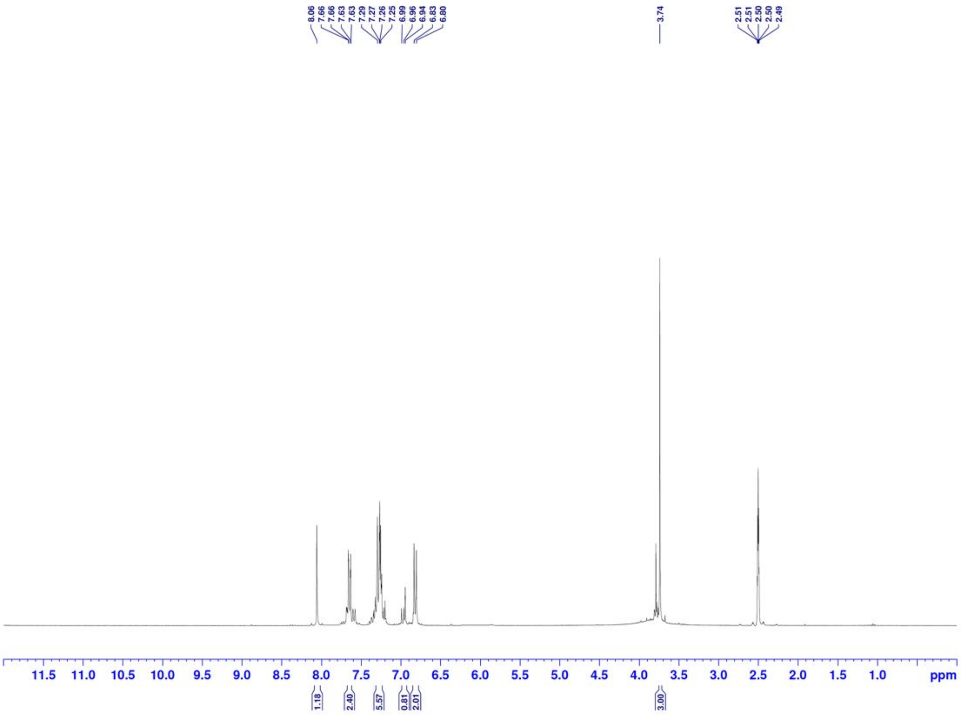

Figure S8. ^{1}H-NMR spectra of compound **2c**

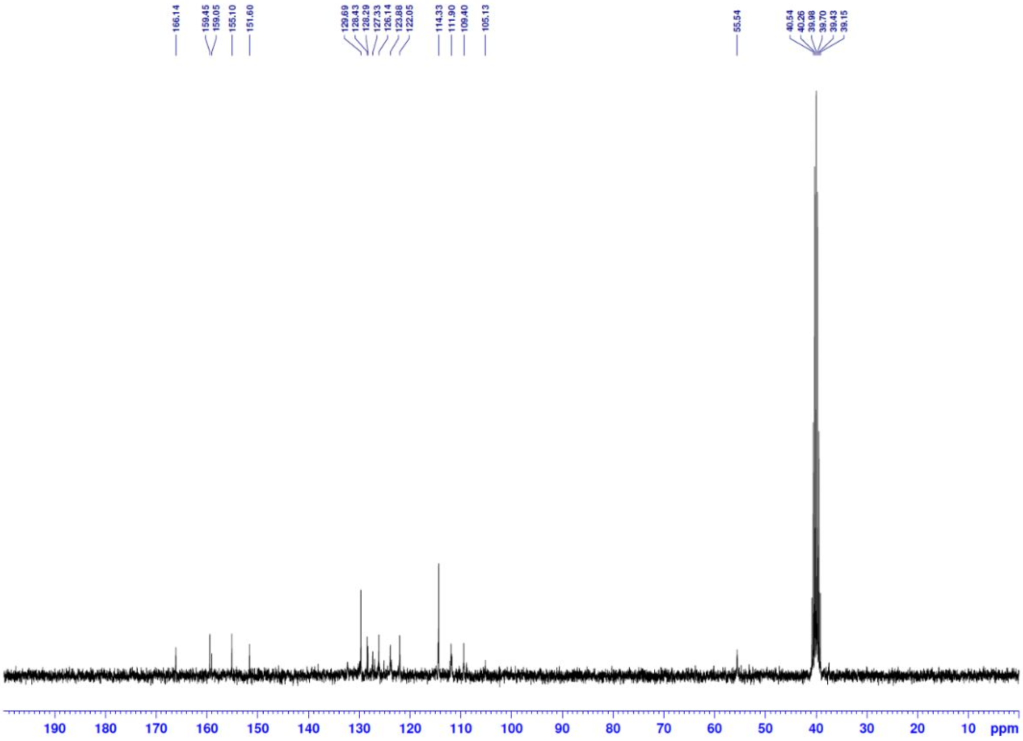

Figure S9. ^{13}C-NMR spectra of compound **2c**

Formula Predictor Report - KD-2d_22.lcd

Data File: C:\LabSolutions\Data\Analiz\derya\KD-2d_22.lcd

Elmt	Val.	Min	Max	Elmt	Val.	Min	Max	Elmt	Val.	Min	Max	Elmt	Val.	Min	Max	Use Adduct
H	1	6	40	O	2	0	5	S	2	1	1	Ru	2	0	0	H
C	4	7	35	F	1	0	0	Cl	1	0	0	Pd	2	0	0	Na
N	3	0	5	P	3	0	0	Br	1	0	0	I	3	0	0	

Error Margin (ppm):	5	DBE Range:	10.0 - 25.0	Electron Ions:	both
HC Ratio:	unlimited	Apply N Rule:	yes	Use MSn Info:	yes
Max Isotopes:	3	Isotope RI (%):	1.00	Isotope Res:	9000
MSn Iso RI (%):	10.00	MSn Logic Mode:	AND	Max Results:	100

Event#: 1 MS(E+) Ret. Time : 1.800 Scan# : 271

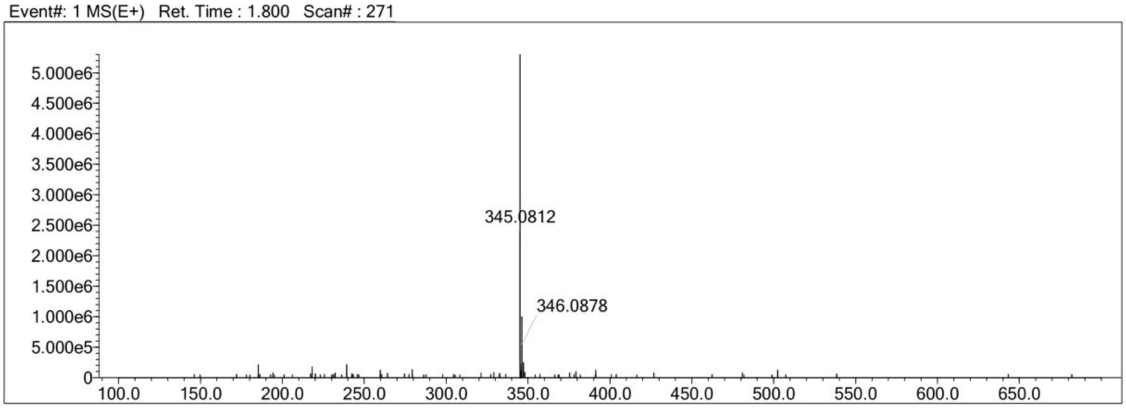

Measured region for 345.0812 m/z

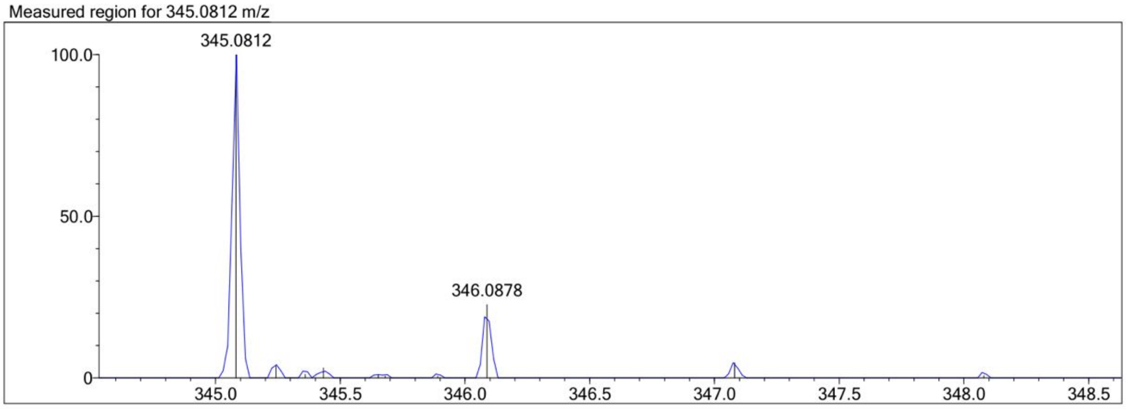

C19 H12 N4 O S [M+H]+ : Predicted region for 345.0805 m/z

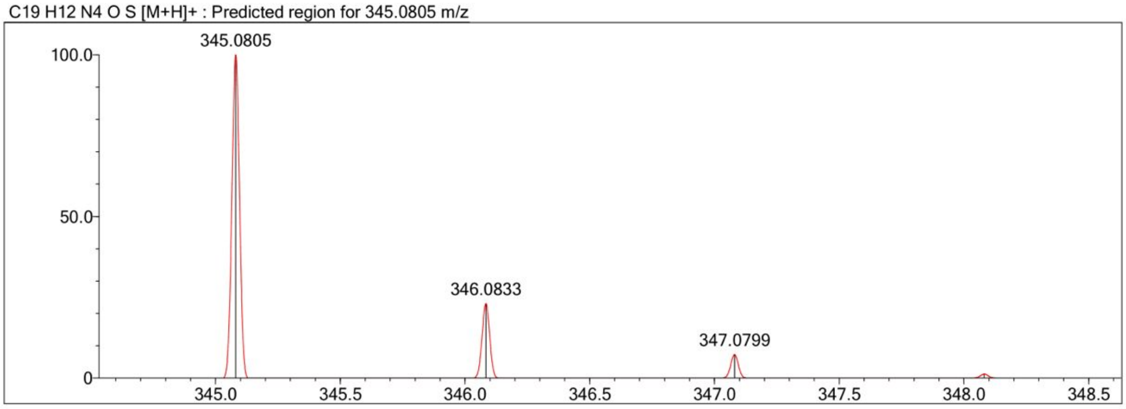

Rank	Score	Formula (M)	Ion	Meas. m/z	Pred. m/z	Df. (mDa)	Df. (ppm)	Iso	DBE
1	67.79	C19 H12 N4 O S	[M+H]+	345.0812	345.0805	0.7	2.03	69.58	16.0

Figure S10. HRMS spectra of compound **2d**

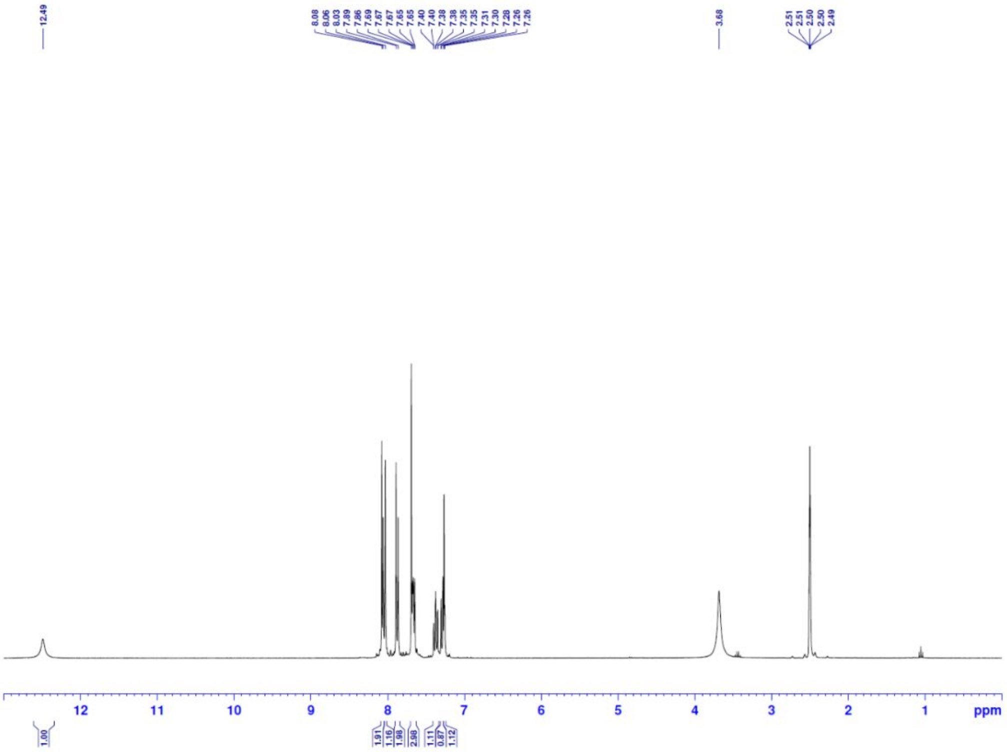

Figure S11. ^{1}H-NMR spectra of compound **2d**

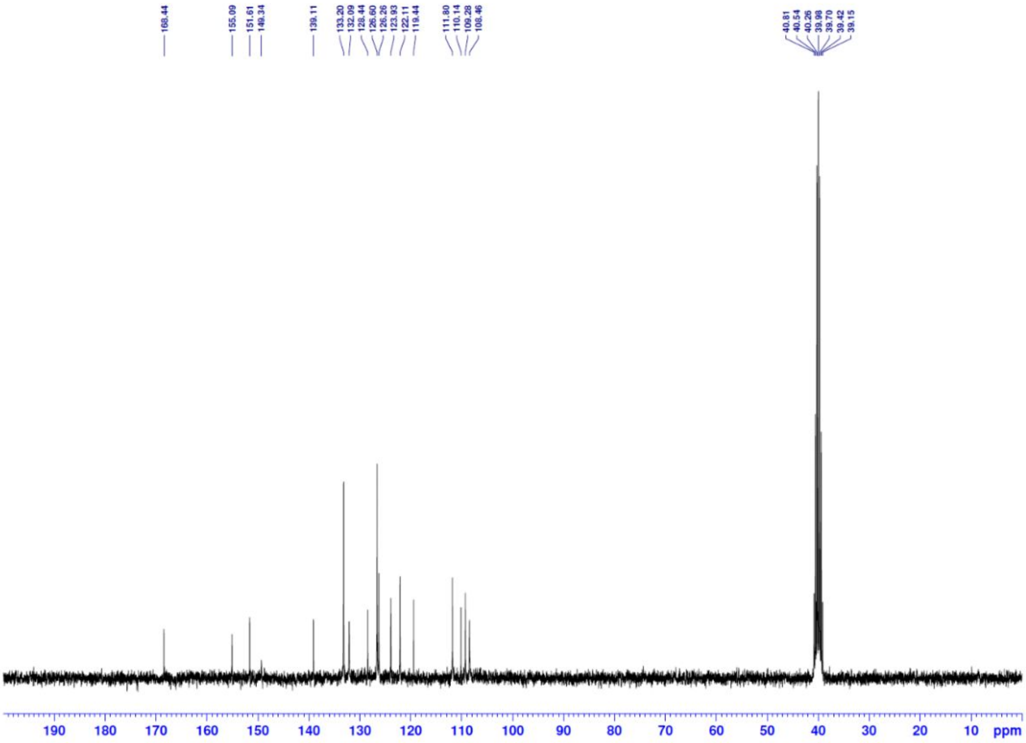

Figure S12. ^{13}C-NMR spectra of compound **2d**

Data File: C:\LabSolutions\Data\Analiz\derya\KD-2e_23.lcd

Elmt	Val.	Min	Max	Elmt	Val.	Min	Max	Elmt	Val.	Min	Max	Elmt	Val.	Min	Max	Use Adduct
H	1	6	40	O	2	0	5	S	2	1	1	Ru	2	0	0	H
C	4	7	35	F	1	0	0	Cl	1	0	0	Pd	2	0	0	Na
N	3	3	5	P	3	0	0	Br	1	0	0	I	3	0	0	

Error Margin (ppm):	5	DBE Range:	10.0 - 25.0	Electron Ions:	both
HC Ratio:	unlimited	Apply N Rule:	yes	Use MSn Info:	yes
Max Isotopes:	3	Isotope RI (%):	1.00	Isotope Res:	9000
MSn Iso RI (%):	10.00	MSn Logic Mode:	AND	Max Results:	100

Event#: 1 MS(E+) Ret. Time : 1.853 Scan# : 279

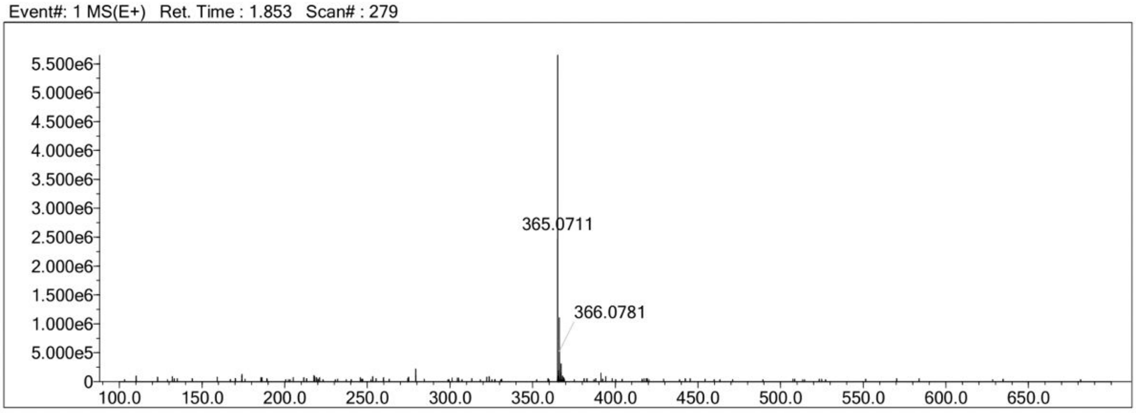

Measured region for 365.0711 m/z

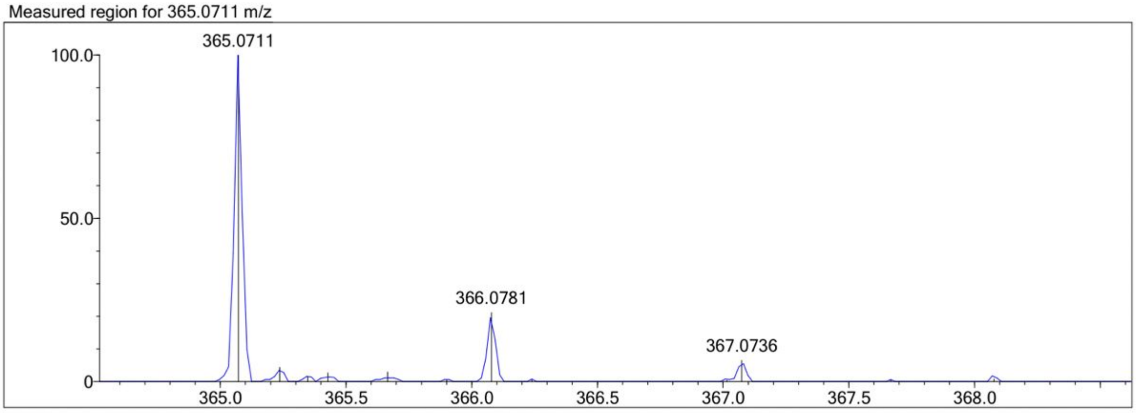

C18 H12 N4 O3 S [M+H]+ : Predicted region for 365.0703 m/z

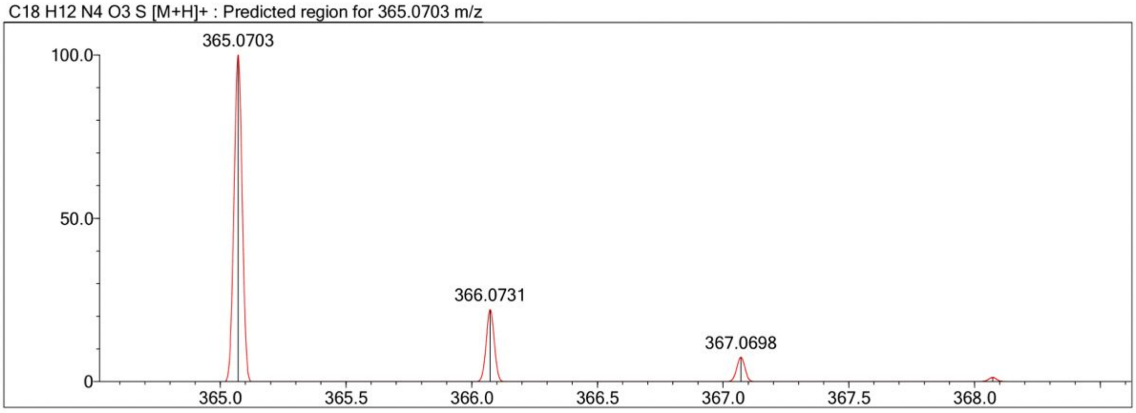

Rank	Score	Formula (M)	Ion	Meas. m/z	Pred. m/z	Df. (mDa)	Df. (ppm)	Iso	DBE
1	77.00	C18 H12 N4 O3 S	[M+H]+	365.0711	365.0703	0.8	2.19	79.36	15.0

Figure S13. HRMS spectra of compound **2e**

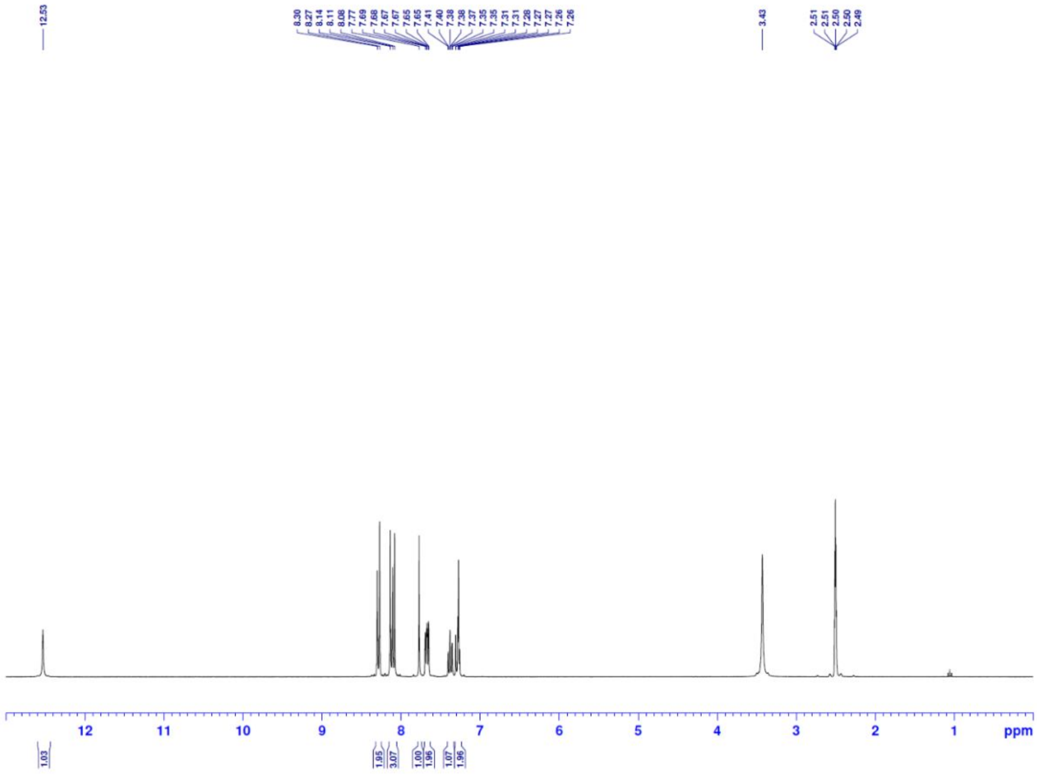

Figure S14. ^{1}H-NMR spectra of compound **2e**

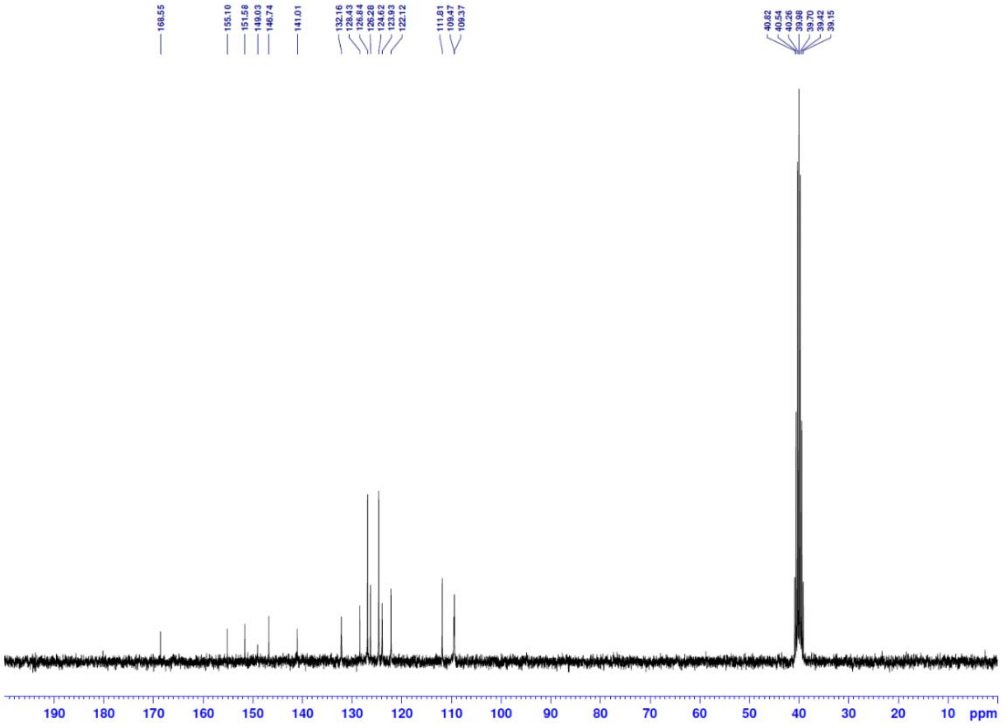

Figure S15. ^{13}C-NMR spectra of compound **2e**

Formula Predictor Report - KD-2f_24.lcd Page 1 of 1

Data File: C:\LabSolutions\Data\Analiz\derya\KD-2f_24.lcd

Elmt	Val.	Min	Max	Elmt	Val.	Min	Max	Elmt	Val.	Min	Max	Elmt	Val.	Min	Max	Use Adduct
H	1	6	40	O	2	0	5	S	2	1	1	Ru	2	0	0	H
C	4	7	35	F	1	1	1	Cl	1	0	0	Pd	2	0	0	Na
N	3	3	5	P	3	0	0	Br	1	0	0	I	3	0	0	

Error Margin (ppm):	5	DBE Range:	10.0 - 25.0	Electron Ions:	both
HC Ratio:	unlimited	Apply N Rule:	yes	Use MSn Info:	yes
Max Isotopes:	3	Isotope RI (%):	1.00	Isotope Res:	9000
MSn Iso RI (%):	10.00	MSn Logic Mode:	AND	Max Results:	100

Event#: 1 MS(E+) Ret. Time : 1.813 Scan# : 273

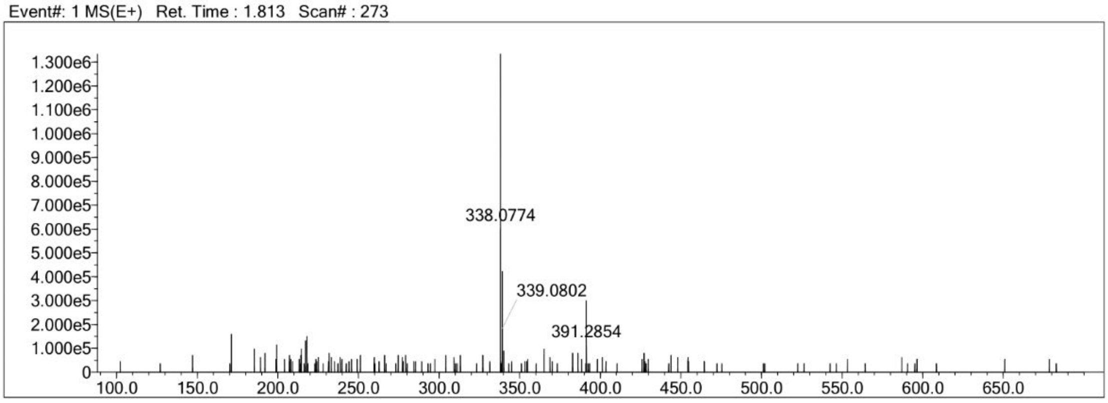

Measured region for 338.0774 m/z

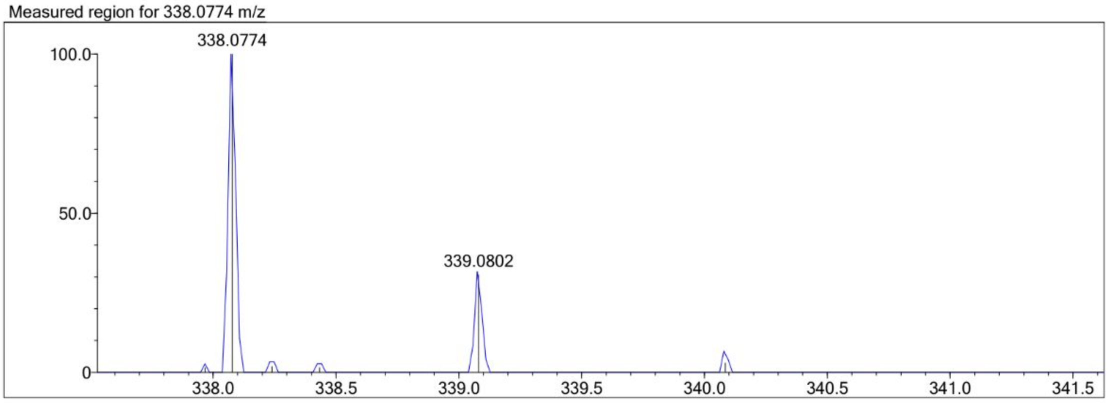

C18 H12 N3 O F S [M+H]+ : Predicted region for 338.0758 m/z

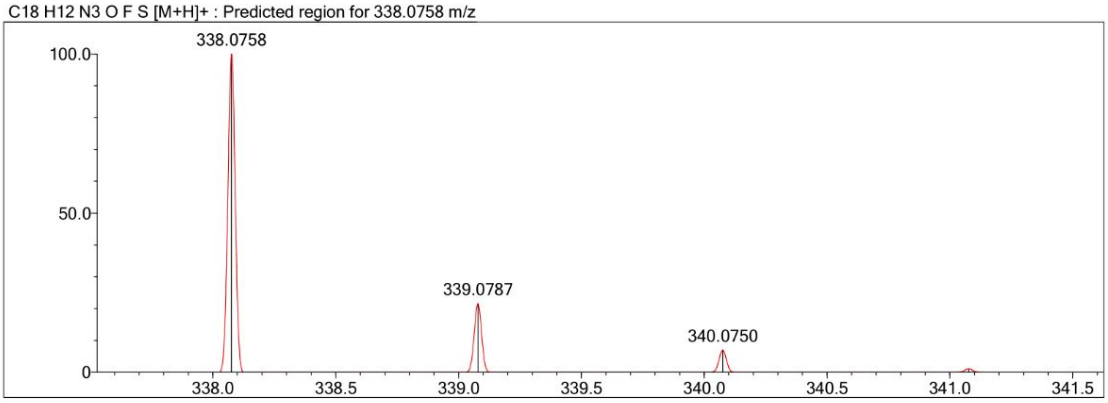

Rank	Score	Formula (M)	Ion	Meas. m/z	Pred. m/z	Df. (mDa)	Df. (ppm)	Iso	DBE
1	43.11	C18 H12 N3 O F S	[M+H]+	338.0774	338.0758	1.6	4.73	47.54	14.0

Figure S16. HRMS spectra of compound **2f**

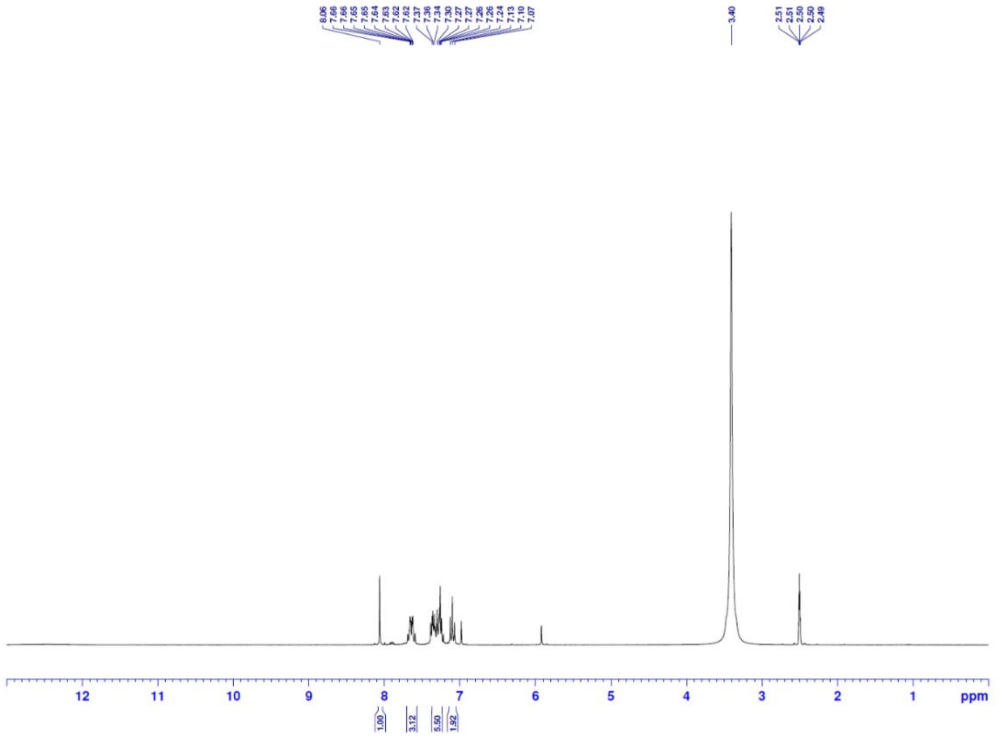

Figure S17. ^{1}H-NMR spectra of compound **2f**

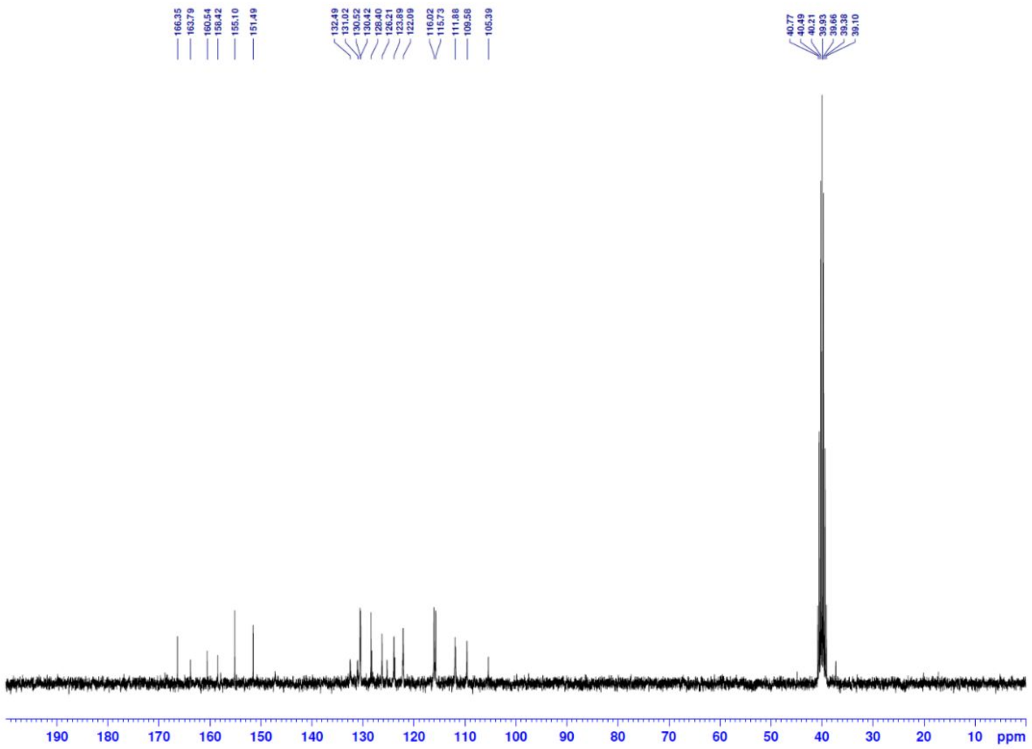

Figure S18. ^{13}C-NMR spectra of compound **2f**

Data File: C:\LabSolutions\Data\Analiz\derya\KD-2g_25.lcd

Elmt	Val.	Min	Max	Elmt	Val.	Min	Max	Elmt	Val.	Min	Max	Elmt	Val.	Min	Max	Use Adduct
H	1	6	40	O	2	0	5	S	2	1	1	Ru	2	0	0	H
C	4	7	35	F	1	0	0	Cl	1	1	1	Pd	2	0	0	Na
N	3	3	5	P	3	0	0	Br	1	0	0	I	3	0	0	

Error Margin (ppm):	5	DBE Range:	10.0 - 25.0	Electron Ions:	both
HC Ratio:	unlimited	Apply N Rule:	yes	Use MSn Info:	yes
Max Isotopes:	3	Isotope RI (%):	1.00	Isotope Res:	9000
MSn Iso RI (%):	10.00	MSn Logic Mode:	AND	Max Results:	100

Event#: 1 MS(E+) Ret. Time : 2.213 Scan# : 333

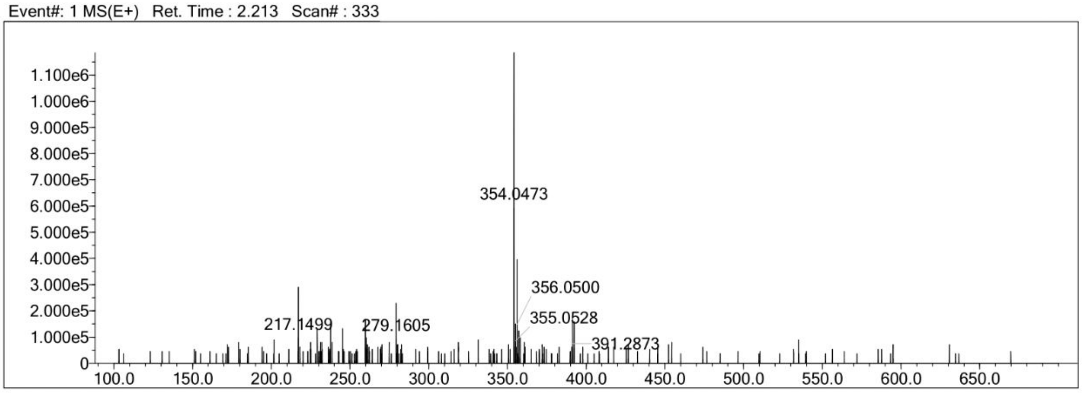

Measured region for 354.0473 m/z

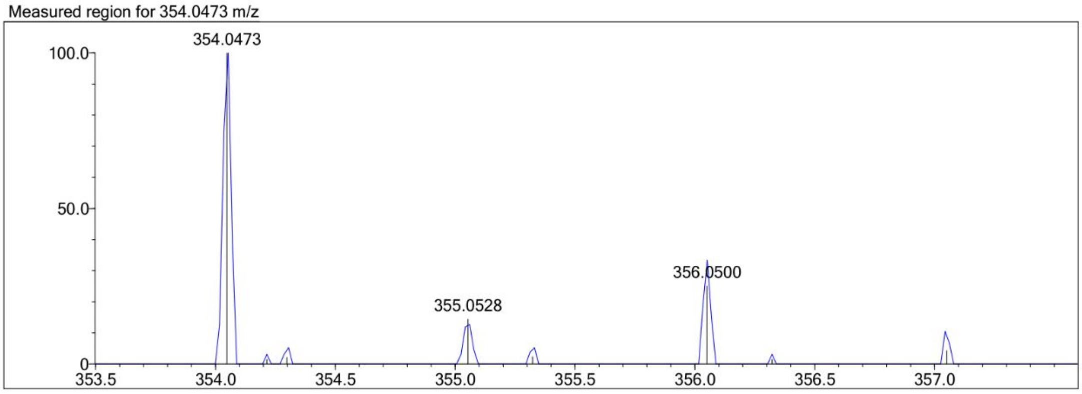

C18 H12 N3 O S Cl [M+H]+ : Predicted region for 354.0462 m/z

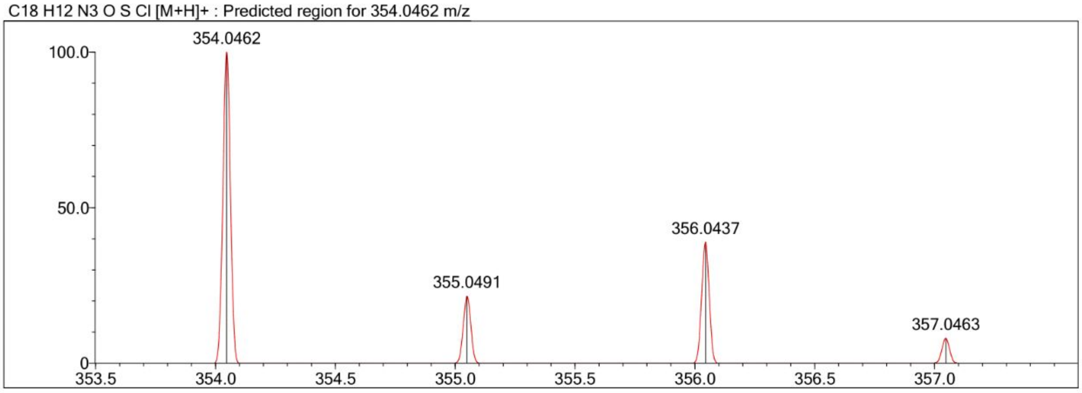

Rank	Score	Formula (M)	Ion	Meas. m/z	Pred. m/z	Df. (mDa)	Df. (ppm)	Iso	DBE
1	48.40	C18 H12 N3 O S Cl	[M+H]+	354.0473	354.0462	1.1	3.11	51.10	14.0

Figure S19. HRMS spectra of compound **2g**

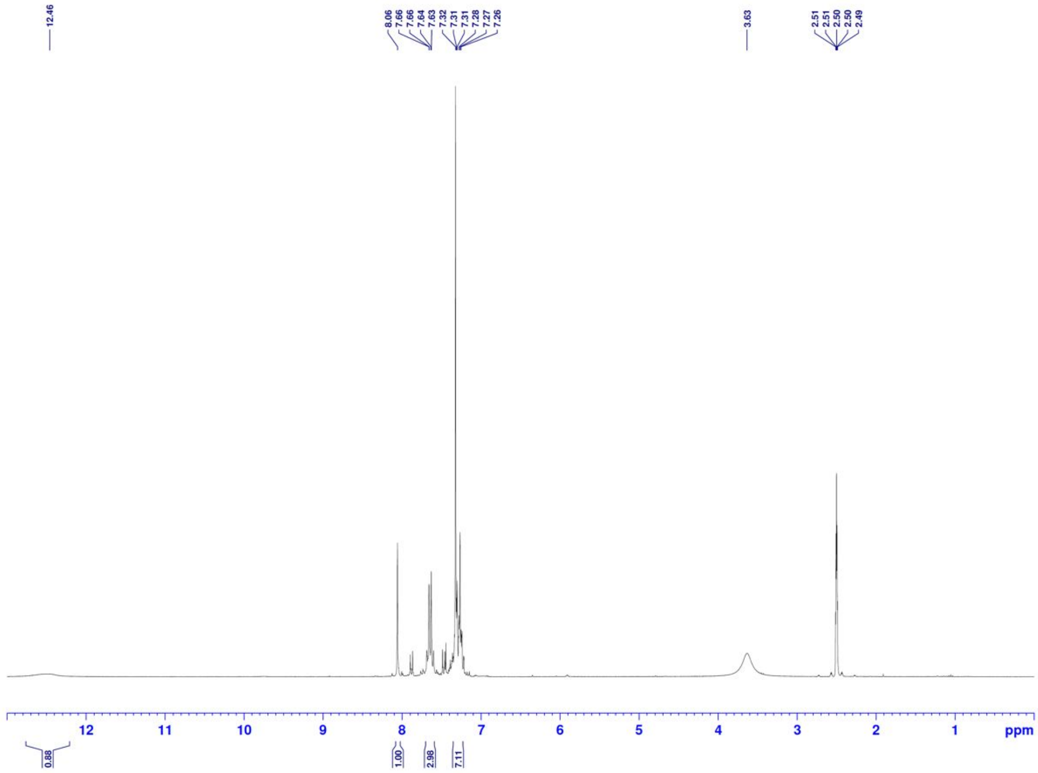

Figure S20. ^{1}H-NMR spectra of compound **2g**

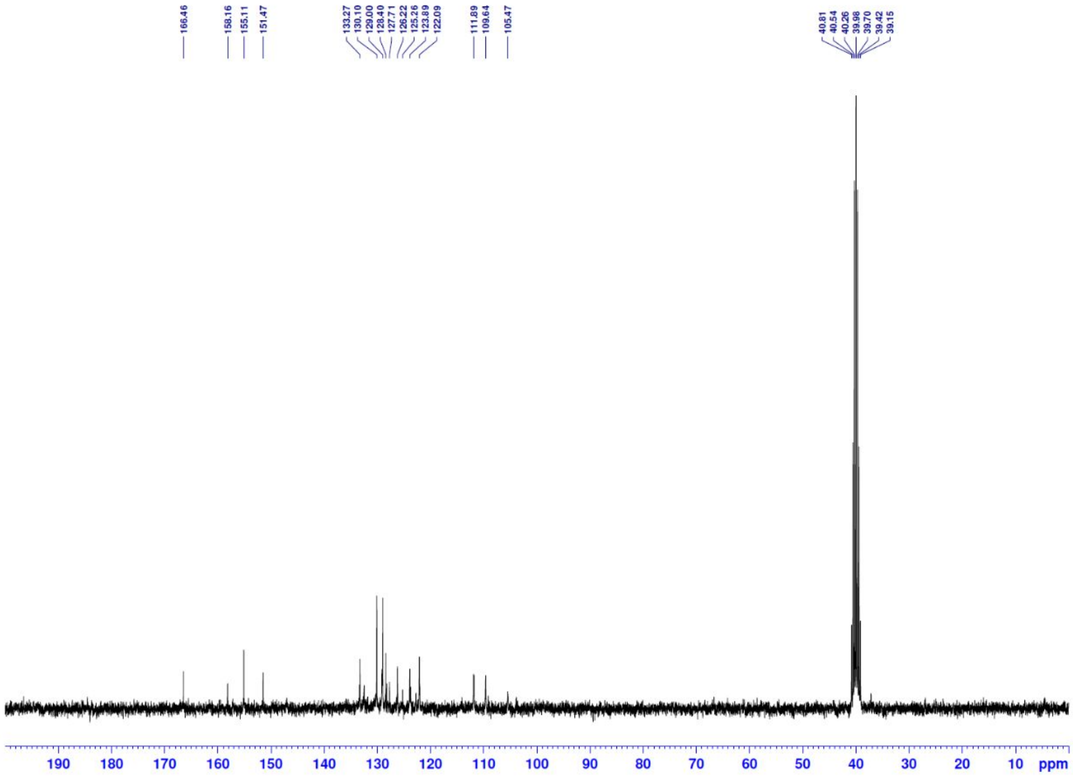

Figure S21. ^{13}C-NMR spectra of compound **2g**

Data File: C:\LabSolutions\Data\Analiz\derya\KD-2h_26.lcd

Elmt	Val.	Min	Max	Elmt	Val.	Min	Max	Elmt	Val.	Min	Max	Elmt	Val.	Min	Max	Use Adduct
H	1	6	40	O	2	0	5	S	2	1	1	Ru	2	0	0	H
C	4	7	35	F	1	0	0	Cl	1	0	0	Pd	2	0	0	Na
N	3	3	5	P	3	0	0	Br	1	1	1	I	3	0	0	

Error Margin (ppm):	5	DBE Range:	10.0 - 25.0	Electron Ions:	both
HC Ratio:	unlimited	Apply N Rule:	yes	Use MSn Info:	yes
Max Isotopes:	3	Isotope RI (%):	1.00	Isotope Res:	9000
MSn Iso RI (%):	10.00	MSn Logic Mode:	AND	Max Results:	100

Event#: 1 MS(E+) Ret. Time : 2.173 Scan# : 327

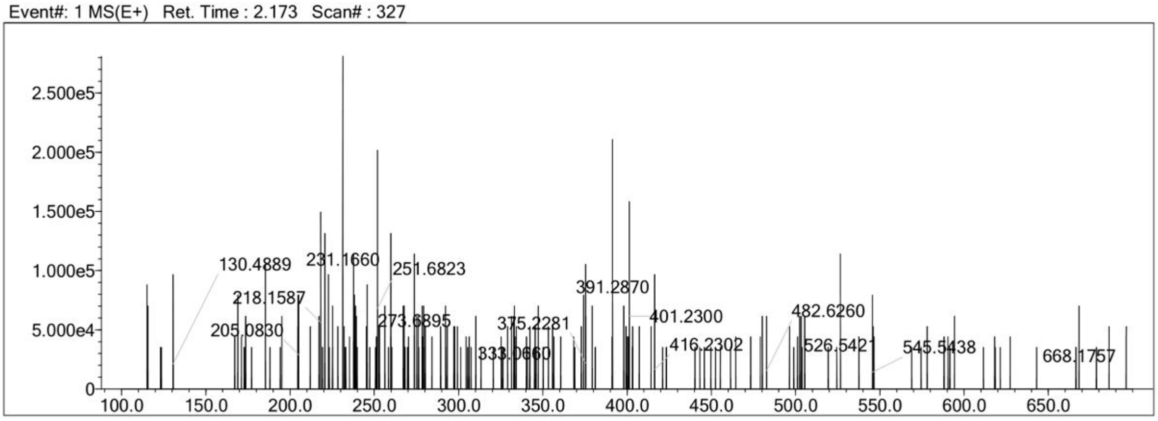

Measured region for 397.9975 m/z

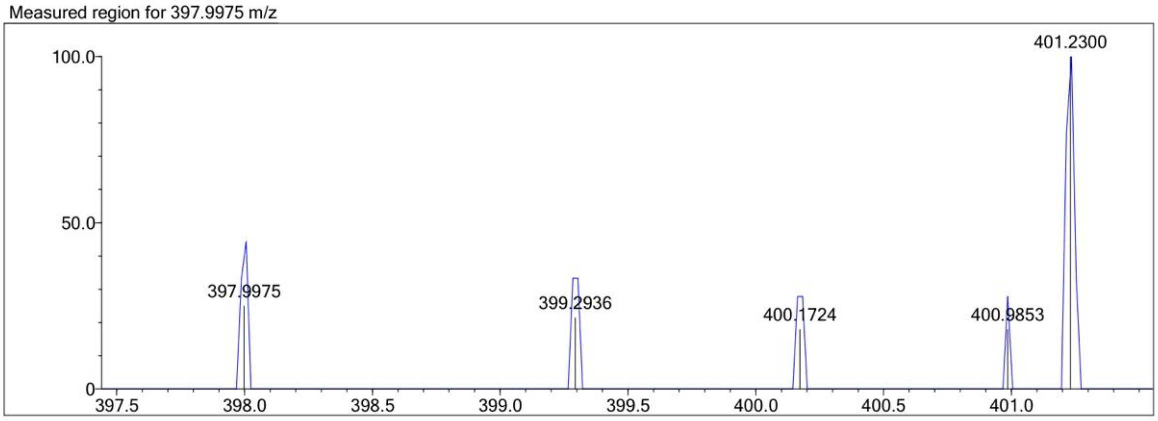

C18 H12 N3 O S Br [M+H]+ : Predicted region for 397.9957 m/z

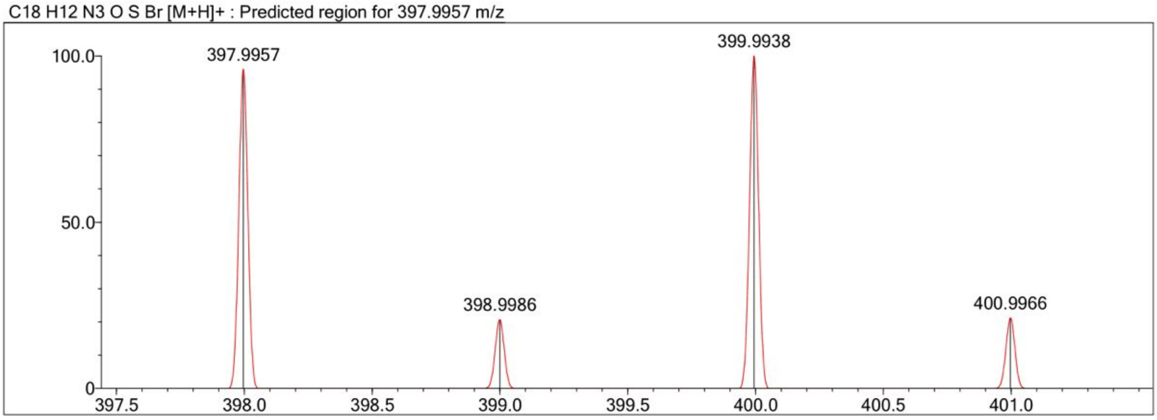

Rank	Score	Formula (M)	Ion	Meas. m/z	Pred. m/z	Df. (mDa)	Df. (ppm)	Iso	DBE
1	0.00	C18 H12 N3 O S Br	[M+H]+	397.9975	397.9957	1.8	4.52	0.00	14.0

Figure S22. HRMS spectra of compound **2h**

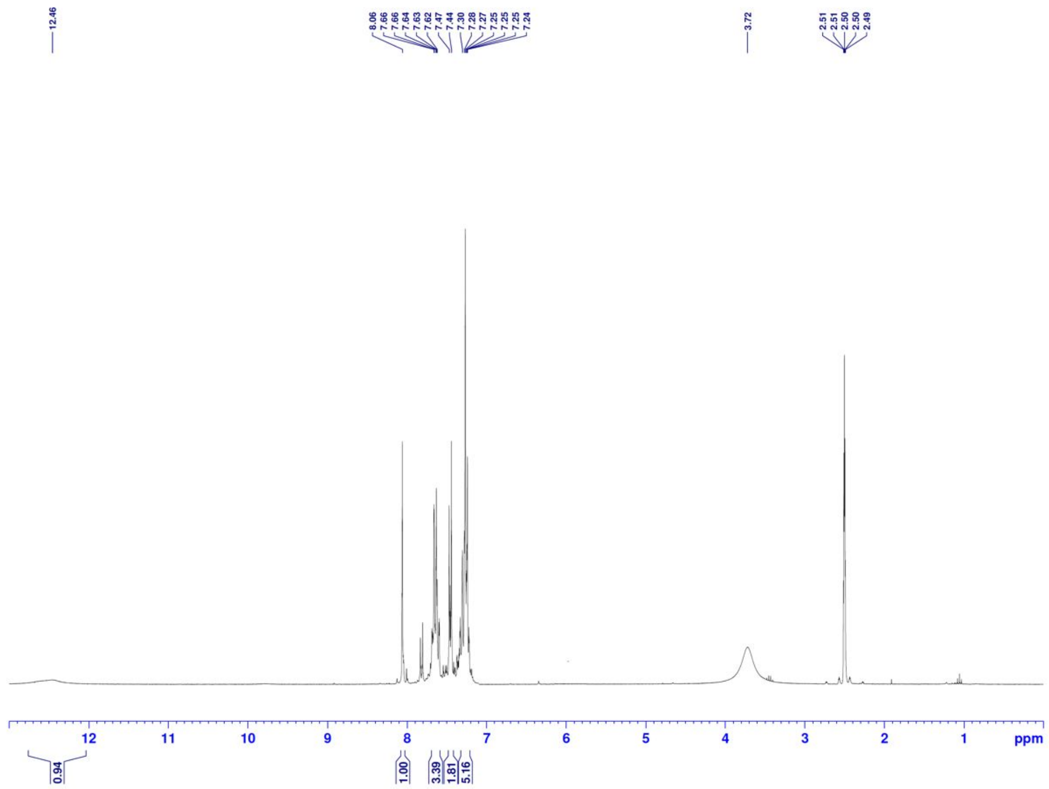

Figure S23. ^{1}H-NMR spectra of compound **2h**

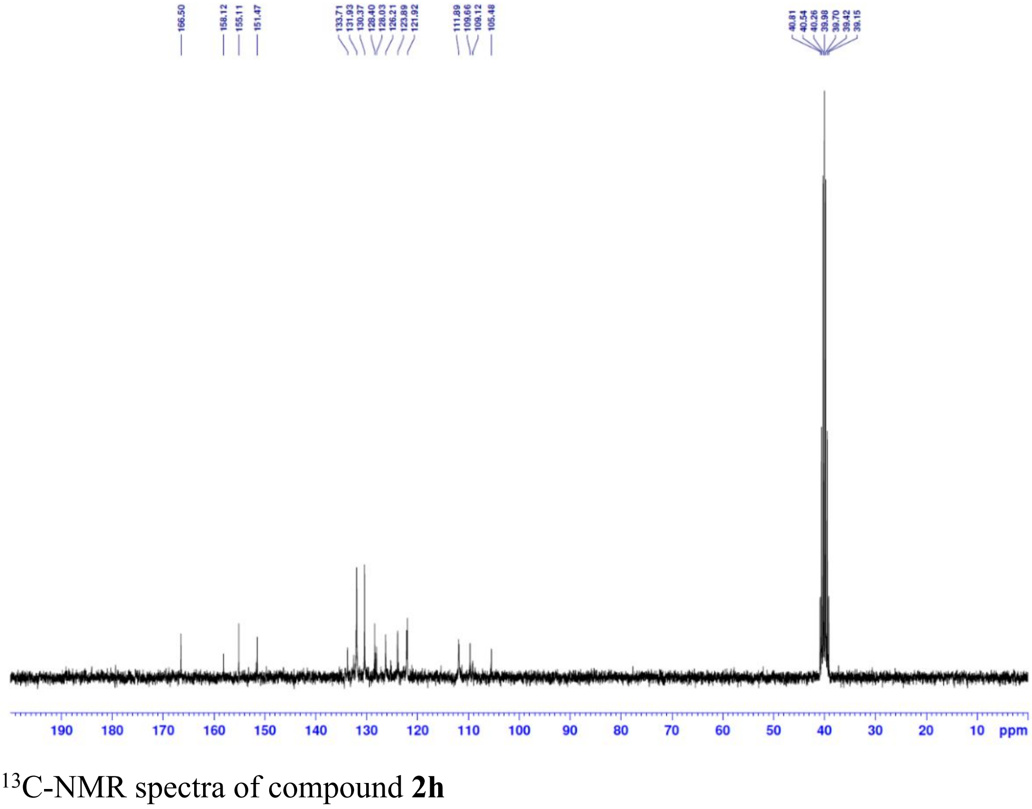

Figure S24. ^{13}C-NMR spectra of compound **2h**

Formula Predictor Report - KD-2i_27.lcd Page 1 of 1

Data File: C:\LabSolutions\Data\Analiz\derya\KD-2i_27.lcd

Elmt	Val.	Min	Max	Elmt	Val.	Min	Max	Elmt	Val.	Min	Max	Elmt	Val.	Min	Max	Use Adduct
H	1	6	40	O	2	0	5	S	2	1	1	Ru	2	0	0	H
C	4	7	35	F	1	0	0	Cl	1	0	0	Pd	2	0	0	Na
N	3	3	5	P	3	0	0	Br	1	0	0	I	3	0	0	

Error Margin (ppm): 5
HC Ratio: unlimited
Max Isotopes: 3
MSn Iso RI (%): 10.00

DBE Range: 10.0 - 25.0
Apply N Rule: yes
Isotope RI (%): 1.00
MSn Logic Mode: AND

Electron Ions: both
Use MSn Info: yes
Isotope Res: 9000
Max Results: 100

Event#: 1 MS(E+) Ret. Time : 2.347 Scan# : 353

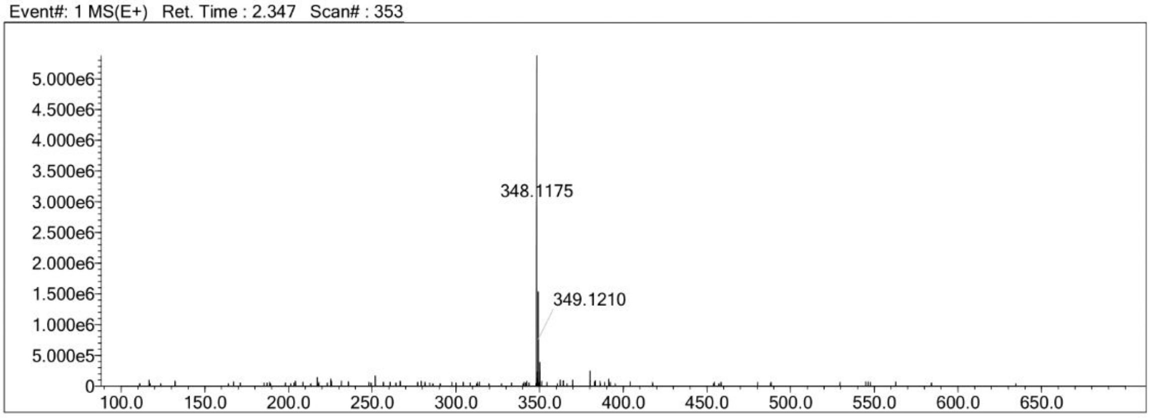

Measured region for 348.1175 m/z

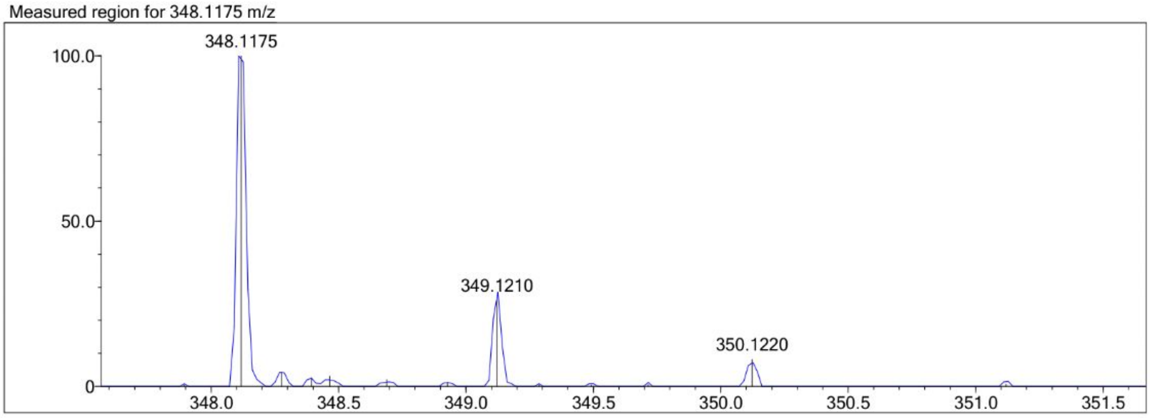

C20 H17 N3 O S [M+H]+ : Predicted region for 348.1165 m/z

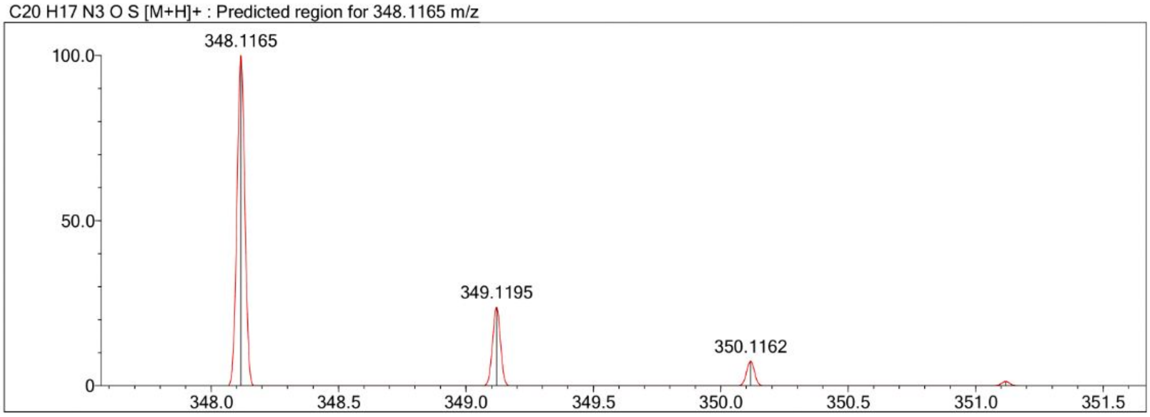

Rank	Score	Formula (M)	Ion	Meas. m/z	Pred. m/z	Df. (mDa)	Df. (ppm)	Iso	DBE
1	87.56	C20 H17 N3 O S	[M+H]+	348.1175	348.1165	1.0	2.87	91.85	14.0

Figure S25. HRMS spectra of compound **2i**

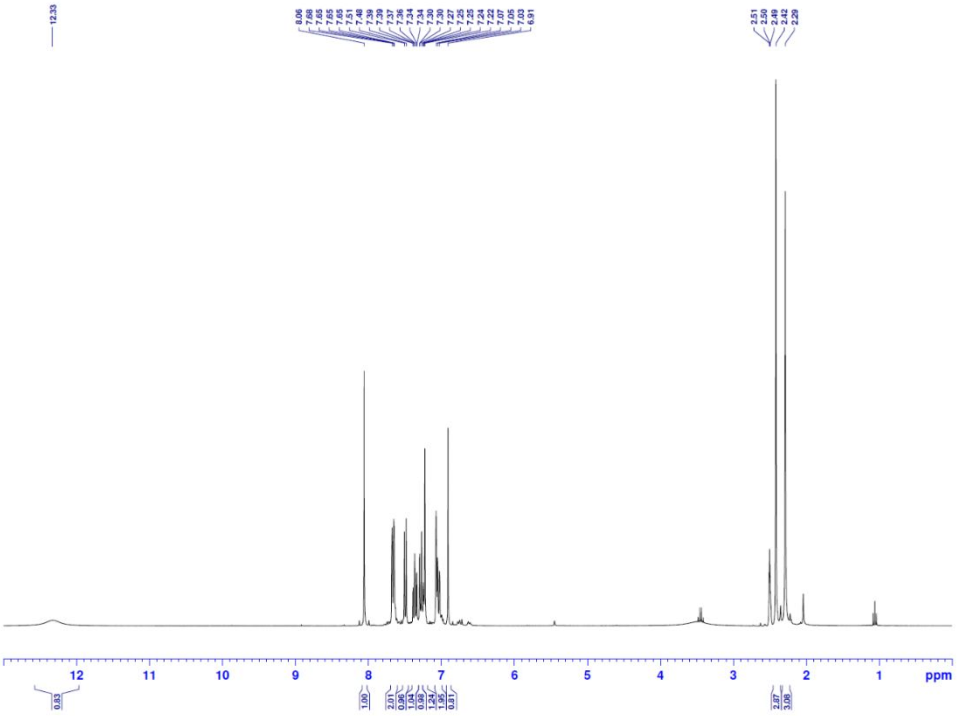

Figure S26. ^{1}H-NMR spectra of compound **2i**

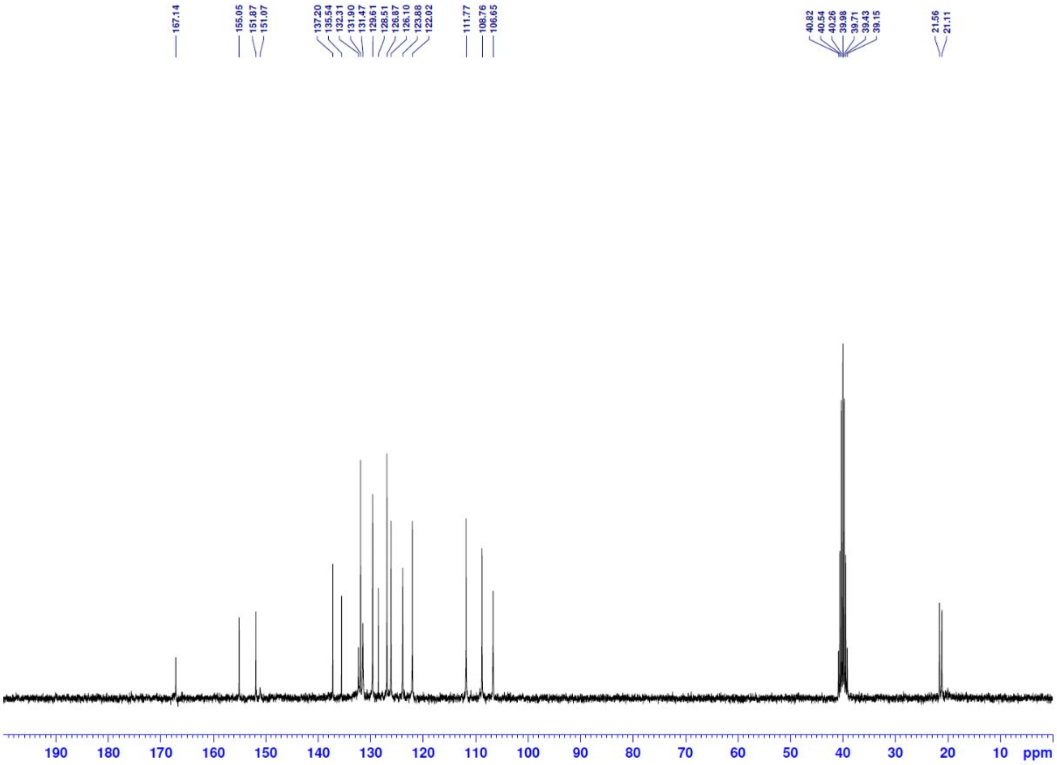

Figure S27. ^{13}C-NMR spectra of compound **2i**

Data File: C:\LabSolutions\Data\Analiz\derya\KD-2j_28.lcd

Elmt	Val.	Min	Max	Elmt	Val.	Min	Max	Elmt	Val.	Min	Max	Elmt	Val.	Min	Max	Use Adduct
H	1	6	40	O	2	0	5	S	2	1	1	Ru	2	0	0	H
C	4	7	35	F	1	0	0	Cl	1	0	0	Pd	2	0	0	Na
N	3	3	5	P	3	0	0	Br	1	0	0	I	3	0	0	

Error Margin (ppm):	5	DBE Range:	10.0 - 25.0	Electron Ions:	both
HC Ratio:	unlimited	Apply N Rule:	yes	Use MSn Info:	yes
Max Isotopes:	3	Isotope RI (%):	1.00	Isotope Res:	9000
MSn Iso RI (%):	10.00	MSn Logic Mode:	AND	Max Results:	100

Event#: 1 MS(E+) Ret. Time : 1.640 Scan# : 247

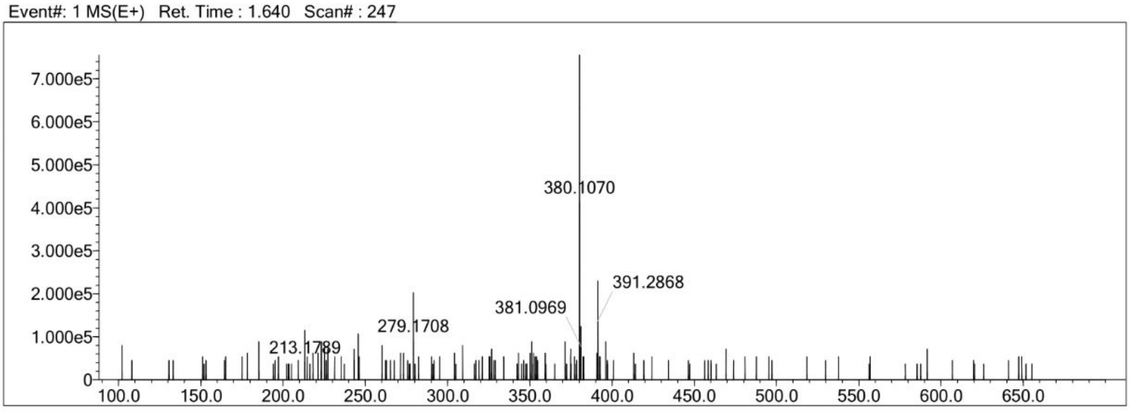

Measured region for 380.1070 m/z

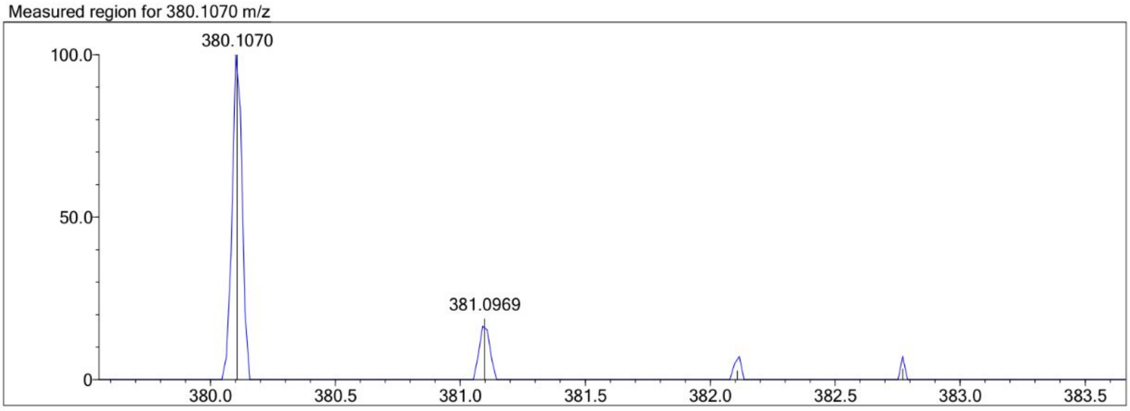

C20 H17 N3 O3 S [M+H]+ : Predicted region for 380.1063 m/z

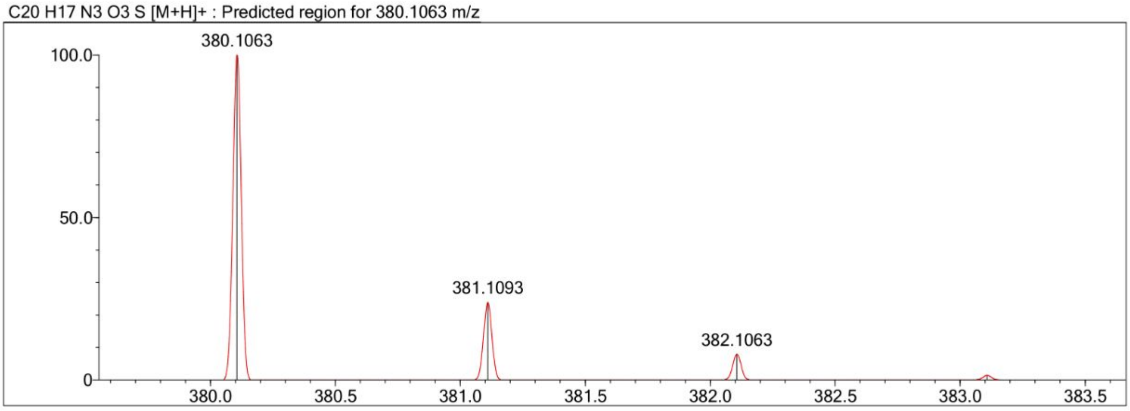

Rank	Score	Formula (M)	Ion	Meas. m/z	Pred. m/z	Df. (mDa)	Df. (ppm)	Iso	DBE
1	44.05	C20 H17 N3 O3 S	[M+H]+	380.1070	380.1063	0.7	1.84	45.00	14.0

Figure S28. HRMS spectra of compound **2j**

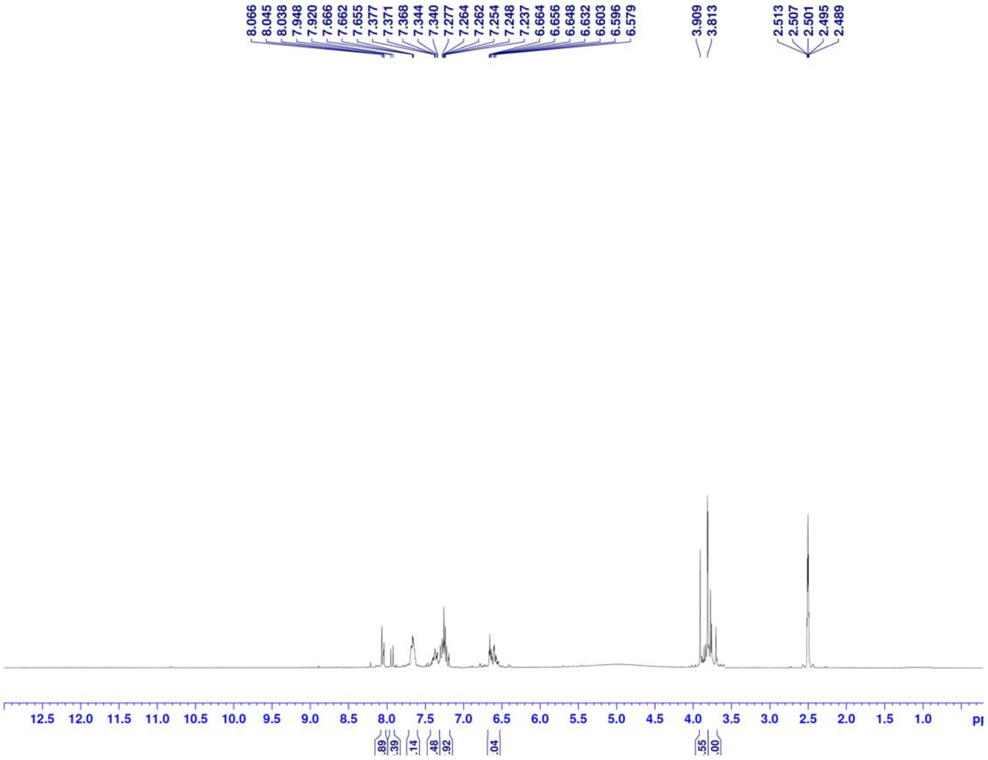

Figure S29. ^{1}H-NMR spectra of compound **2j**

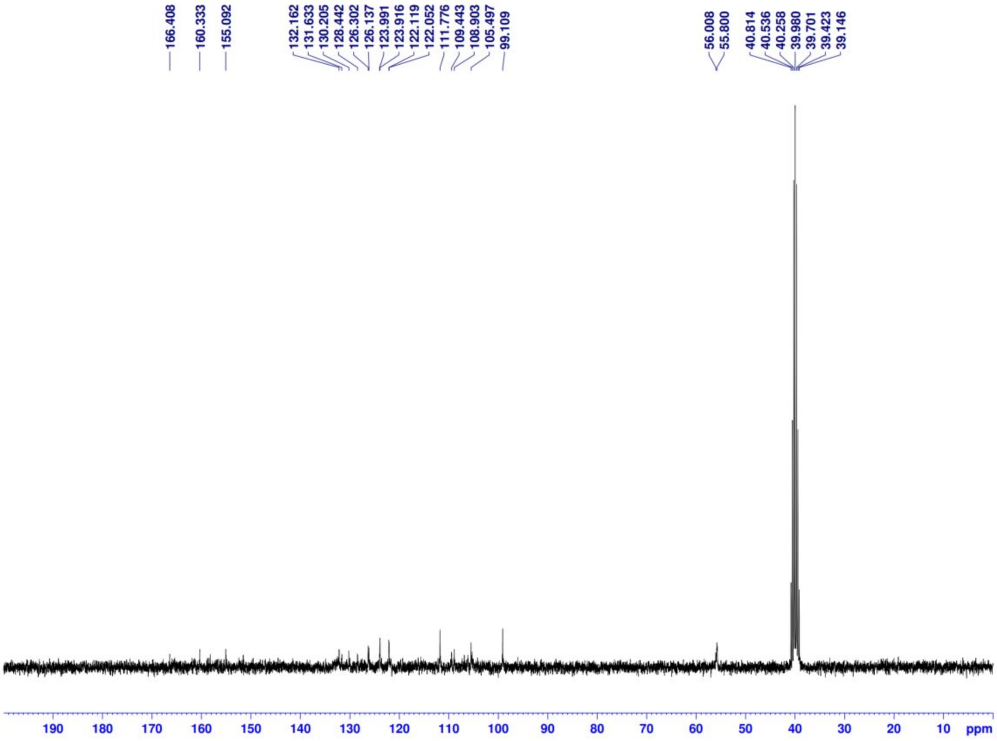

Figure S30. ^{13}C-NMR spectra of compound **2j**

Data File: C:\LabSolutions\Data\Analiz\derya\KD-2k_29.lcd

Elmt	Val.	Min	Max	Elmt	Val.	Min	Max	Elmt	Val.	Min	Max	Elmt	Val.	Min	Max	Use Adduct
H	1	6	40	O	2	0	5	S	2	1	1	Ru	2	0	0	H
C	4	7	35	F	1	2	2	Cl	1	0	0	Pd	2	0	0	Na
N	3	3	5	P	3	0	0	Br	1	0	0	I	3	0	0	

Error Margin (ppm):	5	DBE Range:	10.0 - 25.0	Electron Ions:	both
HC Ratio:	unlimited	Apply N Rule:	yes	Use MSn Info:	yes
Max Isotopes:	3	Isotope RI (%):	1.00	Isotope Res:	9000
MSn Iso RI (%):	10.00	MSn Logic Mode:	AND	Max Results:	100

Event#: 1 MS(E+) Ret. Time : 2.240 Scan# : 337

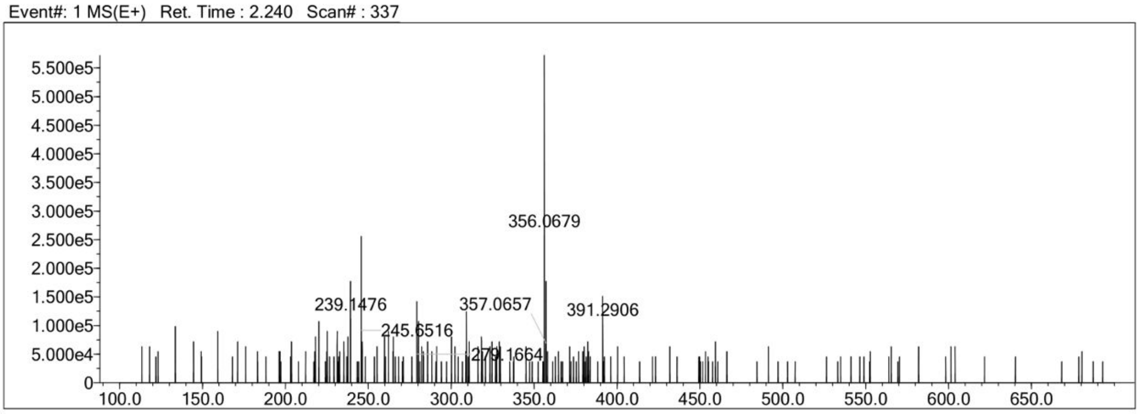

Measured region for 356.0679 m/z

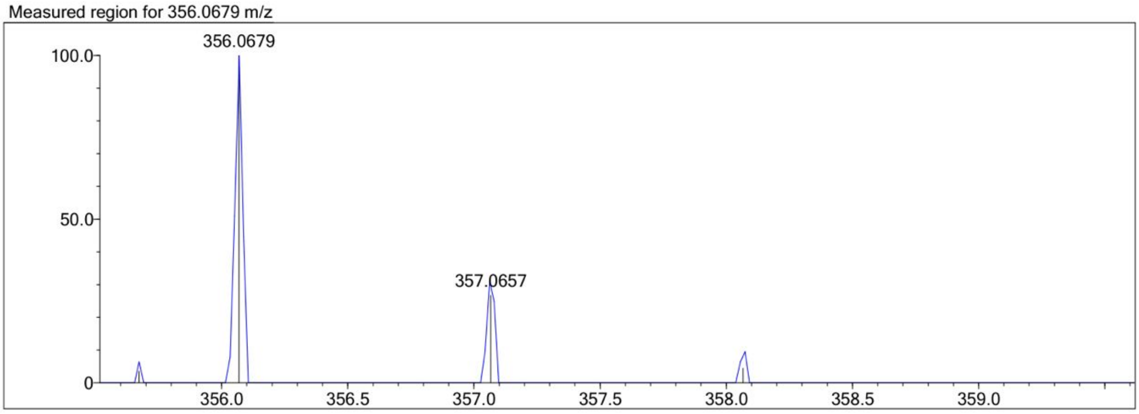

C18 H11 N3 O F2 S [M+H]+ : Predicted region for 356.0664 m/z

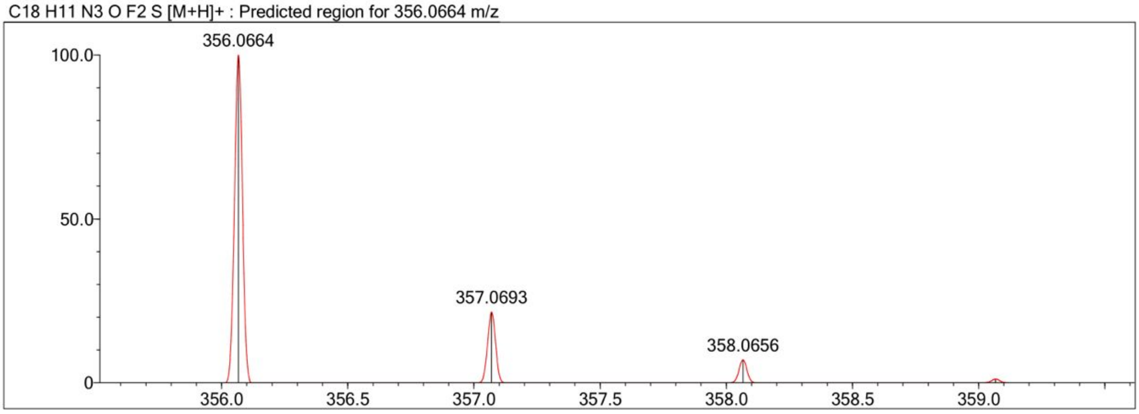

Rank	Score	Formula (M)	Ion	Meas. m/z	Pred. m/z	Df. (mDa)	Df. (ppm)	Iso	DBE
1	53.71	C18 H11 N3 O F2 S	[M+H]+	356.0679	356.0664	1.5	4.21	58.40	14.0

Figure S31. HRMS spectra of compound **2k**

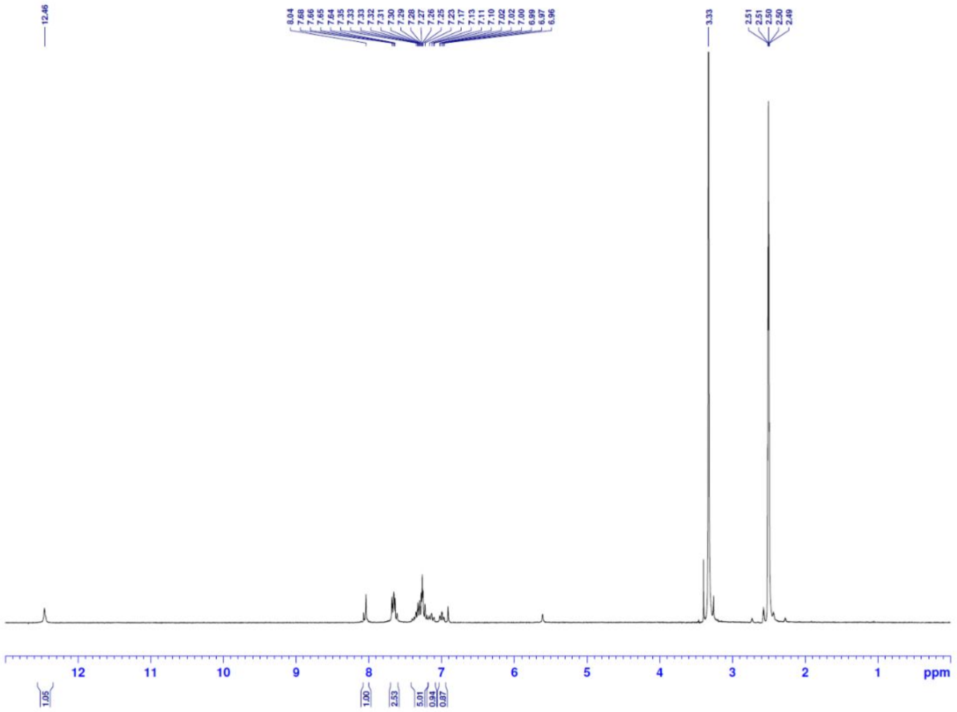

Figure S32. ^{1}H-NMR spectra of compound **2k**

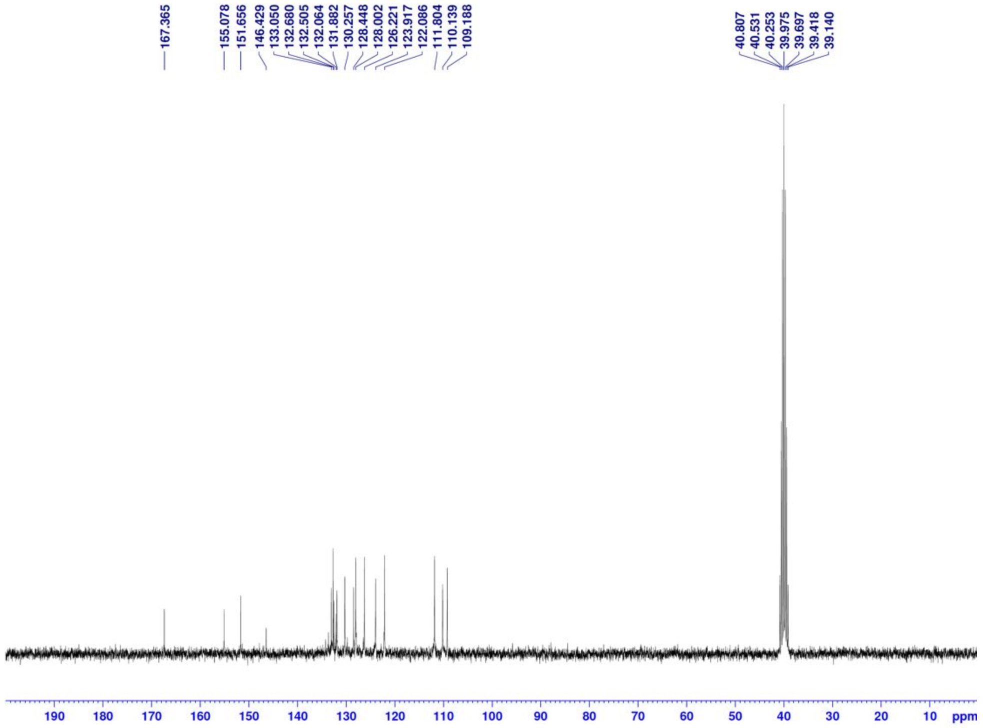

Figure S33. ^{13}C-NMR spectra of compound **2k**

Formula Predictor Report - KD-2l_30.lcd — Page 1 of 1

Data File: C:\LabSolutions\Data\Analiz\derya\KD-2l_30.lcd

Elmt	Val.	Min	Max	Elmt	Val.	Min	Max	Elmt	Val.	Min	Max	Elmt	Val.	Min	Max	Use Adduct
H	1	6	40	O	2	0	5	S	2	1	1	Ru	2	0	0	H
C	4	7	35	F	1	0	0	Cl	1	2	2	Pd	2	0	0	Na
N	3	3	5	P	3	0	0	Br	1	0	0	I	3	0	0	

Error Margin (ppm):	5	DBE Range:	10.0 - 25.0	Electron Ions:	both
HC Ratio:	unlimited	Apply N Rule:	yes	Use MSn Info:	yes
Max Isotopes:	3	Isotope RI (%):	1.00	Isotope Res:	9000
MSn Iso RI (%):	10.00	MSn Logic Mode:	AND	Max Results:	100

Event#: 1 MS(E+) Ret. Time : 4.427 Scan# : 665

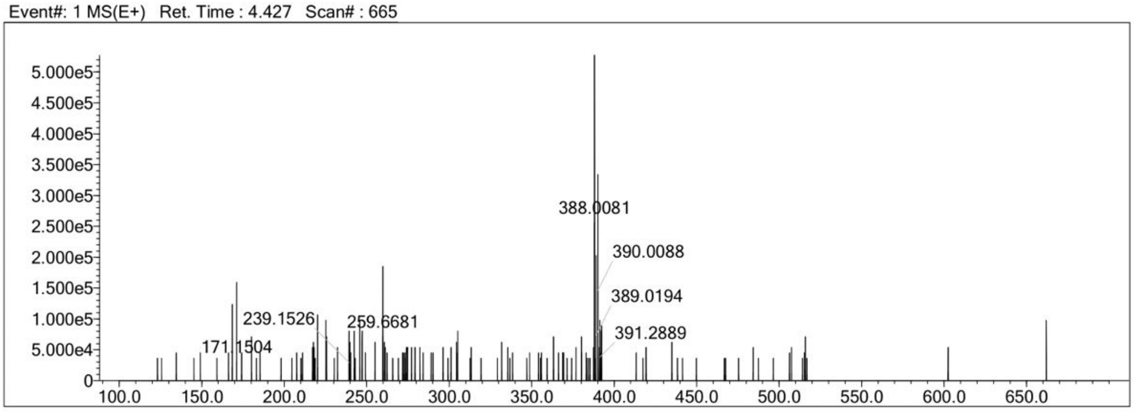

Measured region for 388.0081 m/z

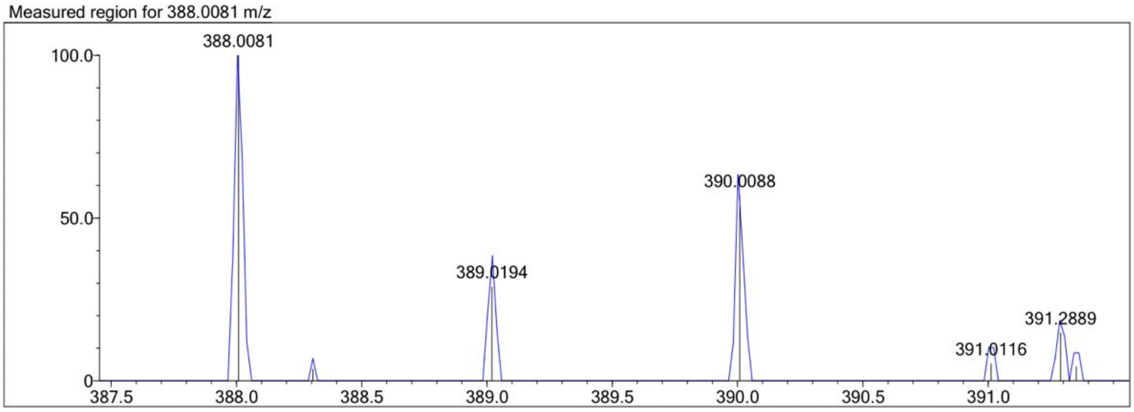

C18 H11 N3 O S Cl2 [M+H]+ : Predicted region for 388.0073 m/z

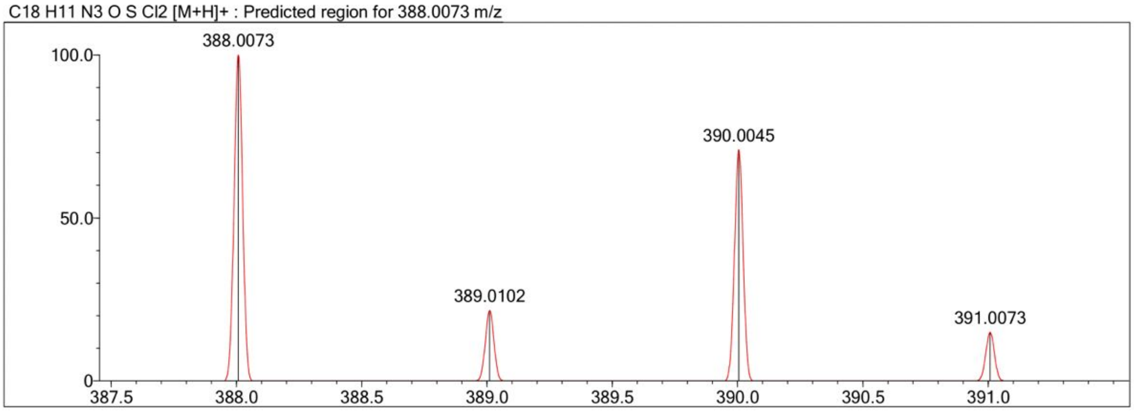

Rank	Score	Formula (M)	Ion	Meas. m/z	Pred. m/z	Df. (mDa)	Df. (ppm)	Iso	DBE
1	45.49	C18 H11 N3 O S Cl2	[M+H]+	388.0081	388.0073	0.8	2.06	46.72	14.0

Figure S34. HRMS spectra of compound **2l**

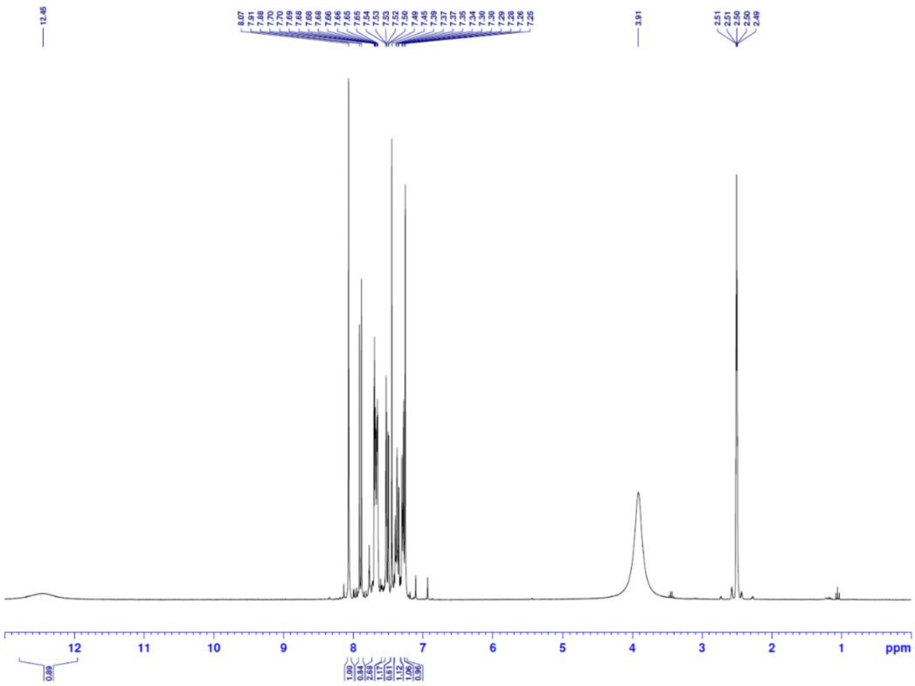

Figure S35. ^{1}H-NMR spectra of compound **2l**

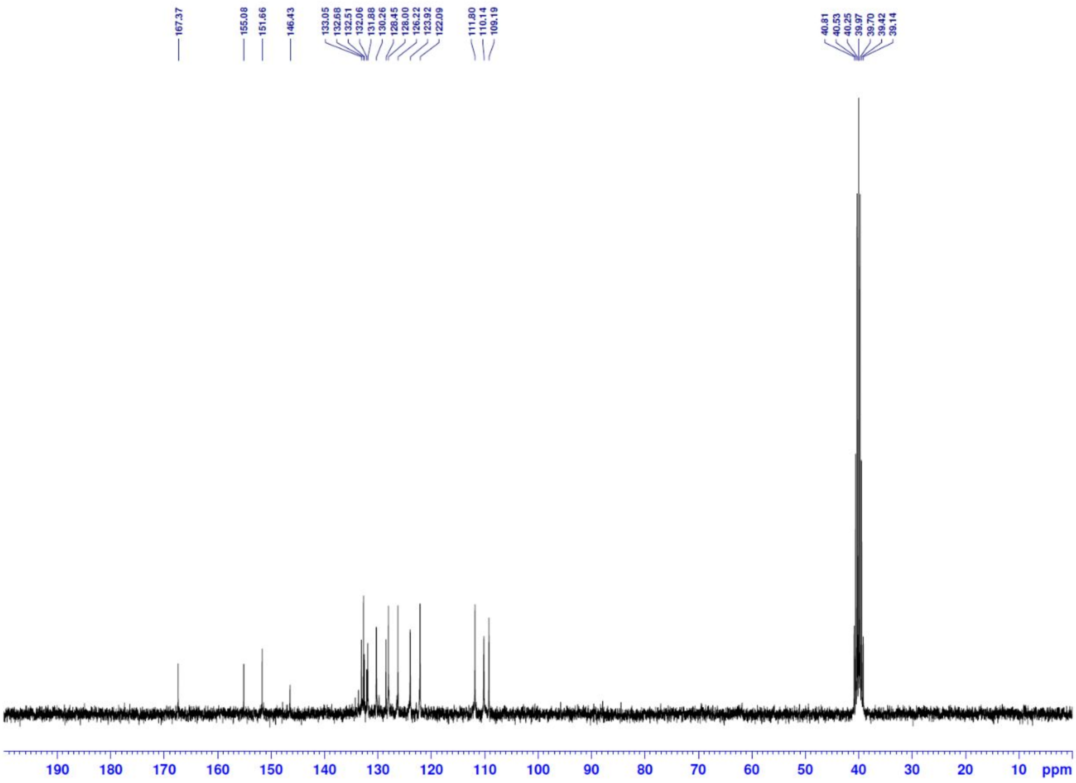

Figure S36. ^{13}C-NMR spectra of compound **2l**
